# A framework to identify contributing genes in patients with Phelan-McDermid syndrome

**DOI:** 10.1038/s41525-017-0035-2

**Published:** 2017-10-23

**Authors:** Anne-Claude Tabet, Thomas Rolland, Marie Ducloy, Jonathan Lévy, Julien Buratti, Alexandre Mathieu, Damien Haye, Laurence Perrin, Céline Dupont, Sandrine Passemard, Yline Capri, Alain Verloes, Séverine Drunat, Boris Keren, Cyril Mignot, Isabelle Marey, Aurélia Jacquette, Sandra Whalen, Eva Pipiras, Brigitte Benzacken, Sandra Chantot-Bastaraud, Alexandra Afenjar, Delphine Héron, Cédric Le Caignec, Claire Beneteau, Olivier Pichon, Bertrand Isidor, Albert David, Laila El Khattabi, Stephan Kemeny, Laetitia Gouas, Philippe Vago, Anne-Laure Mosca-Boidron, Laurence Faivre, Chantal Missirian, Nicole Philip, Damien Sanlaville, Patrick Edery, Véronique Satre, Charles Coutton, Françoise Devillard, Klaus Dieterich, Marie-Laure Vuillaume, Caroline Rooryck, Didier Lacombe, Lucile Pinson, Vincent Gatinois, Jacques Puechberty, Jean Chiesa, James Lespinasse, Christèle Dubourg, Chloé Quelin, Mélanie Fradin, Hubert Journel, Annick Toutain, Dominique Martin, Abdelamdjid Benmansour, Claire S. Leblond, Roberto Toro, Frédérique Amsellem, Richard Delorme, Thomas Bourgeron

**Affiliations:** 1 Genetics Department, Robert Debré Hospital, APHP, Paris, France; 2 Human Genetics and Cognitive Functions, Institut Pasteur, Paris, France; 3 CNRS UMR 3571 Genes, Synapses and Cognition, Institut Pasteur, Paris, France; 4 Sorbonne Paris Cité, Human Genetics and Cognitive Functions, Université Paris Diderot, Paris, France; 5 Cytogenetics Unit, Pitié Salpetrière Hospital, APHP, Paris, France; 6 Neurogenetics Unit, Pitié Salpetrière Hospital, APHP, Paris, France; 7 Clinical Genetics Unit, Pitié Salpetrière Hospital, APHP, Paris, France; 8 Cytogenetics Unit, Jean Verdier Hospital, APHP, Bondy, France; 9 Cytogenetics Unit, Trousseau Hospital, APHP, Paris, France; 10 Clinical Genetics Unit, Trousseau Hospital, APHP, Paris, France; 11 Clinical Genetics Unit, Nantes Hospital, Nantes, France; 12 Cytogenetics Unit, Cochin Hospital, APHP, Paris, France; 13 Genetics Unit, CHU Estaing, Clermont-Ferrand, France; 14 Cytogenetics Unit, Dijon Hospital, Dijon, France; 15 Clinical Genetics Unit, Dijon Hospital, Dijon, France; 16 Genetics Unit, La Timone Hospital, Marseille, France; 17 Cytogenetics Unit, Lyon Civil Hospital, Lyon, France; 18 Clinical Genetics Unit, Lyon Civil Hospital, Lyon, France; 19 Cytogenetics Unit, Grenoble Hospital, Grenoble, France; 20 Clinical Genetics Unit, Grenoble Hospital, Grenoble, France; 21 Genetics Unit, Bordeaux Hospital, Bordeaux, France; 22 Genetics Unit, Montpellier Hospital, Montpellier, France; 23 Genetics Unit, CHRU Nimes, Nimes, France; 24 Cytogenetics Unit, Chambéry-Hôtel-Dieu Hospital, Chambéry, France; 25 Genetics Unit, CHU Rennes, Rennes, France; 26 Genetics Unit, Chubert Hospital, Vannes, France; 27 Genetics Unit, Bretonneau Hospital, Tours, France; 28 Genetics Unit, CH Le Mans, Le Mans, France; 29 Department of Child and Adolescent Psychiatry, Robert Debré Hospital, APHP, Paris, France

**Keywords:** Clinical genetics, Neurodevelopmental disorders

## Abstract

Phelan-McDermid syndrome (PMS) is characterized by a variety of clinical symptoms with heterogeneous degrees of severity, including intellectual disability (ID), absent or delayed speech, and autism spectrum disorders (ASD). It results from a deletion of the distal part of chromosome 22q13 that in most cases includes the *SHANK3* gene. *SHANK3* is considered a major gene for PMS, but the factors that modulate the severity of the syndrome remain largely unknown. In this study, we investigated 85 patients with different 22q13 rearrangements (78 deletions and 7 duplications). We first explored the clinical features associated with PMS, and provide evidence for frequent corpus callosum abnormalities in 28% of 35 patients with brain imaging data. We then mapped several candidate genomic regions at the 22q13 region associated with high risk of clinical features, and suggest a second locus at 22q13 associated with absence of speech. Finally, in some cases, we identified additional clinically relevant copy-number variants (CNVs) at loci associated with ASD, such as 16p11.2 and 15q11q13, which could modulate the severity of the syndrome. We also report an inherited *SHANK3* deletion transmitted to five affected daughters by a mother without ID nor ASD, suggesting that some individuals could compensate for such mutations. In summary, we shed light on the genotype-phenotype relationship of patients with PMS, a step towards the identification of compensatory mechanisms for a better prognosis and possibly treatments of patients with neurodevelopmental disorders.

## The underlying genetics of Phelan-McDermid syndrome

Multiple chromosomal changes may impact the severity of symptoms in people with Phelan-McDermid syndrome (PMS). Thomas Bourgeron of the Institut Pasteur and colleagues in France conducted genomic analyses and explored the clinical features of 85 people with PMS, a condition caused by a deletion in the long arm of chromosome 22. It is often associated with severe symptoms such as intellectual disability, autism, and seizures. The chromosomal changes in 65% of those studied were not inherited. MRI brain scans showed visible abnormalities in 23 of 35 patients imaged. The size of the chromosomal deletion varied. Patients with small deletions were more likely to have autistic symptoms while those with large deletions were more likely to be unable to talk. The team also identified genes in chromosome 22 and in other regions of the genome that could modify the severity of PMS symptoms.

## Introduction

Phelan-McDermid syndrome (PMS) is a severe genetic condition characterized by hypotonia, global developmental delay, intellectual disability (ID), absent or delayed speech, minor dysmorphic features, and autism spectrum disorders (ASD).^1^ It results from a deletion of the 22q13 region in the distal part of the long arm of chromosome 22. PMS occurs in 0.2–0.4% of individuals with neurodevelopmental disorders (NDD),^2,3^ but the prevalence remains difficult to ascertain due to important biases in the identification of the patients.

In most reported cases, the 22q13 deletion appeared de novo.^4^ The size of the deleted genomic segment varies from hundreds of kilobases (kb) to more than nine megabases (Mb). The mechanisms resulting in the deletion are also highly variable, including simple deletions, unbalanced translocations, ring chromosomes or more complex chromosomal rearrangements.^2,3^ In the vast majority of the cases, the deletion includes *SHANK3*, a gene associated with several neuropsychiatric disorders including ID, ASD and schizophrenia.^5–7^ Rare interstitial deletions at 22q13 not encompassing *SHANK3* were also observed in patients with features of PMS, suggesting the implication of additional genes in that region or a positional effect influencing *SHANK3* expression.^8,9^

*SHANK3* codes for a scaffolding protein at the post-synaptic density of glutamatergic synapses, and is known to play a critical role in synaptic function by modulating the formation of dendrites.^10,11^ Mice lacking *Shank3* present alterations in the morphogenesis of the dendritic spines of hippocampal neurons and abnormal synaptic protein levels at the post-synaptic density of glutamatergic synapses.^12^ Studies using human neurons derived from induced pluripotent stem cells of patients with PMS revealed that abnormal networks are reversible by an overexpression of the SHANK3 protein or by treatment with insulin growth factor 1 (IGF1).^13^ *SHANK3* mutations can also cause a channelopathy due to a reduction of the hyperpolarization-activated cyclic nucleotide-gated channel proteins (HCN proteins).^14^ Finally, using iPSCs from patients with ASD and carrying de novo* SHANK3* loss-of-function mutations, Darville et al. identified lithium as a factor increasing *SHANK3* transcripts.^15^

Patients diagnosed with PMS display a large range of clinical features with various degrees of functional impact. Most patients are dependent on their caregivers. Associated medical or psychiatric comorbidities include seizures (grand mal seizures, focal seizures and absence seizures), renal abnormalities (mainly absent kidney, structural abnormalities of the kidney, hydronephrosis and kidney reflux), cardiac defects (tricuspid valve regurgitation, atrial septal defect, patent ductus arteriosus, and total anomalous pulmonary return), gastrointestinal disorders (intestinal/anal atresia, chronic constipation, gastroesophageal reflux), and ophthalmic features (most often strabismus).^16^ The molecular basis for this clinical heterogeneity is still largely unresolved. To date, at least 13 case series have been published gathering 584 affected patients with PMS.^16^ Only five studies (including 310 subjects) investigated the correlation of clinical features variability and the size of the 22q13 deletion,^17–21^ but the causality remains unclear. In addition, none of these studies explored the role of additional genetic variants (“multiple-hits”) in the diversity and severity of the symptoms reported in patients.^22,23^

In the present study, we collected clinical and genomic data from 85 patients carrying an unbalanced genomic rearrangement involving *SHANK3* (78 deletions and 7 duplications). We first describe the impact of the size of the 22q13 deletion on the clinical features. Then, we report the identification of multiple-hits in patients with PMS, some affecting known ASD-risk loci that could influence the diversity and severity of the phenotype. Finally, we illustrate the clinical and genomic heterogeneity of individuals carrying 22q13 rearrangements in a family with five affected siblings who inherited a *SHANK3* deletion from a mother without ID nor ASD, providing a proof of principle that some individuals could compensate for such mutations.

## Results

### The 22q13 rearrangements: prevalence and clinical features

**Fig. 1:**
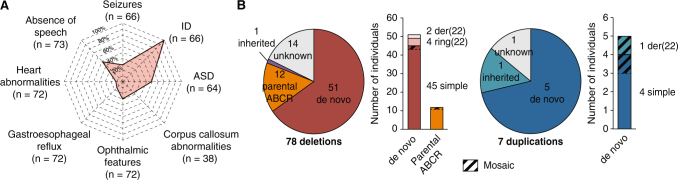
Clinical and genetic description of the cohort. **a** Spider plot of the clinical profile of the patients with PMS as a percentage of cases having each feature over those tested. **b** Mechanisms and inheritance of identified 22q13 CNVs in the cohort. *ABCR* apparently balanced chromosomal rearrangement, *der(22)* derived from unbalanced reciprocal translocation, *ring(22)* caused by a ring chromosome 22

Based on the results of seven clinical centers gathering 18 115 patients with ID, ASD and/or congenital malformations, we could provide a broad estimation of 0.27% for the prevalence of 22q13 deletions among patients with NDD (0.21 to 1.38% depending on the clinical center) (Supplementary Table 1). Unlike most NDD for which males are significantly more affected than females, we observed a balanced sex ratio with 37 males and 39 females carrying a 22q13 deletion. Clinical features of patients included in this study were similar to those previously reported in larger datasets of patients (Fig. 1, Supplementary Information). However, for 35 patients, we analyzed the structural brain magnetic resonance imaging (MRI) data and 23 had macroscopic anomalies (65.7%), consistent with those already described in PMS (Supplementary Information). Remarkably, ten of these patients (28%) presented abnormalities of the corpus callosum ranging from thinness to agenesis (Supplementary Fig. 1) indicating that this abnormality is found in a relatively large subgroup of patients with PMS.

### The 22q13 rearrangements: inheritance and sizes

Our cohort included 78 patients carrying a deletion at 22q13 overlapping *SHANK3* and seven patients with a duplication (Supplementary Tables 2, 3). The deletions mostly derived from simple de novo deletions (57%), including two that were mosaic in the somatic cells of the patients (Fig. 1). Other de novo chromosomal rearrangements included terminal deletions caused by a ring chromosome 22 in four cases, and deletions derived from unbalanced reciprocal translocations in two cases. Altogether, de novo rearrangements accounted for 51 cases (65.4%). In 12 cases, the deletions resulted from recombined parental balanced translocations or inversions. The size of the deleted segment was highly variable, ranging from 45.8 kb to 9.1 Mb (Supplementary Fig. 2).

The duplications occurred de novo in 5 cases. For P10, it was inherited from a healthy mother and for P61, inheritance was unknown. Two duplications were in mosaic (P29 and P50), including one case (P29) where the duplication resulted from an unbalanced translocation. The size of the duplicated segment ranged from 96 kb to 5.8 Mb.

### Exploratory cluster analysis of PMS clinical heterogeneity

**Fig. 2:**
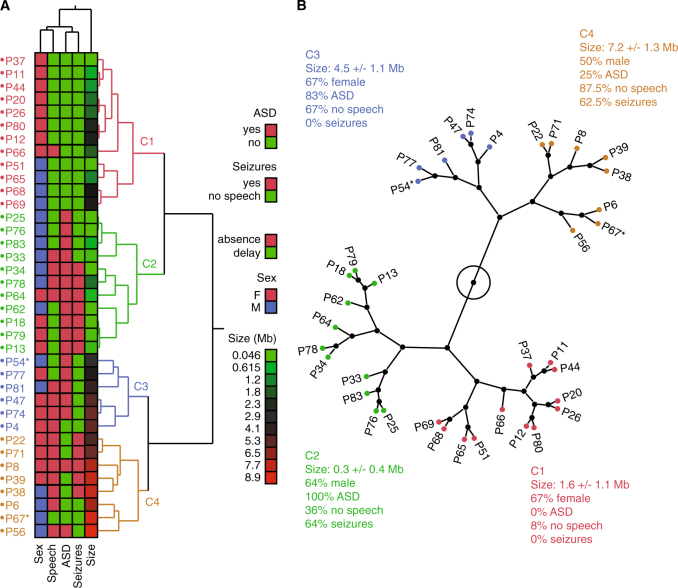
Multivariate analysis of the 22q13 deletion size, sex and clinical features of patients. **a** Hierarchical clustering based on multivariate analysis shows four clusters. **b** Tree view showing the four clusters of patients and the main genomic and clinical features of each cluster. * indicate patients carrying mosaic CNVs

To first explore the phenotypic and genetic heterogeneity of the PMS patients, we performed a hierarchical clustering analysis on 37 individuals for whom information concerning sex and presence or absence of ASD traits, speech and seizures were available. Four clusters summarized the variability (Fig. 2). The main separation was based on the size of the deletion. Clusters C1 (12 patients) and C2 (11 patients) had deletions of small size (0.3–1.6 Mb), but differed for ASD and seizures (C1: 0% ASD and 0% seizures; C2: 100% ASD and 64% seizures). Clusters C3 (six patients) and C4 (eight patients) were characterized by large deletions of an average of 4.5 and 7.2 Mb, respectively. These two clusters showed very similar percentages of patients with absence of speech (>65% in both clusters), but differed specifically for seizures with 0 and 62.5% of the patients in cluster C3 and C4, respectively. Therefore, the size of the 22q13 deletion might at least partly explain the presence and severity of the PMS symptoms. These results based on a subset of 37 patients with all phenotypic data available prompted a detailed and statistically robust mapping of the genomic regions associated to each specific symptom in the entire cohort in order to identify the contributing genes.

### Association between clinical features and genomic regions at 22q13

**Fig. 3:**
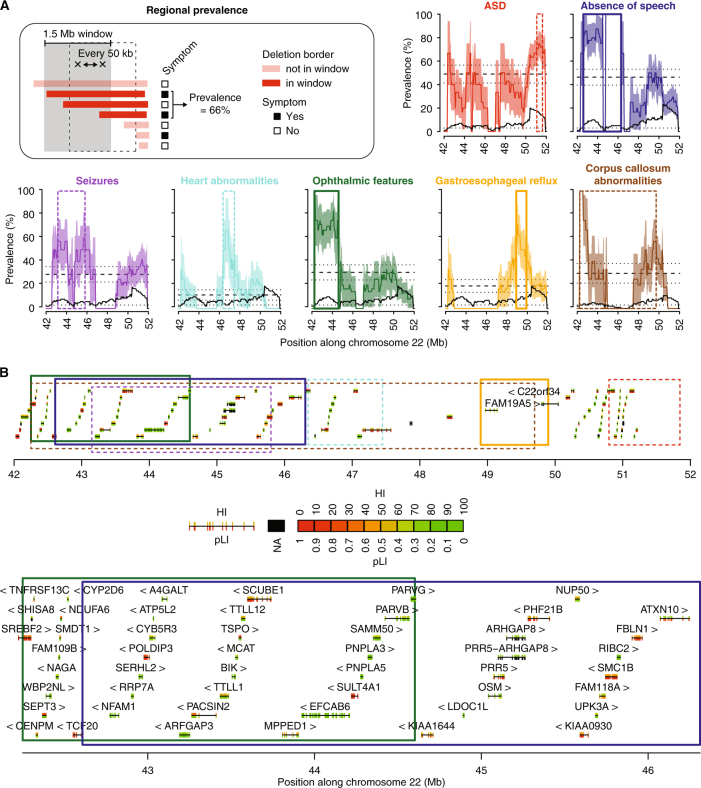
Mapping of genomic regions at 22q13 associated with high risk of presenting clinical features. **a** Prevalence is measured each 50 kb, within overlapping windows of 1.5 Mb (Materials and Methods). Dashed lines represent the global prevalence of each feature, measured as the fraction of patients with 22q13 deletions presenting the feature over all patients with 22q13 deletions. Dotted lines and colored areas represent standard errors of the proportion. Black solid lines show the numbers of informative patients for each window, and black dotted lines correspond to the minimum number of individuals required by window (*n* = 3) to reduce interpretation biases. **b** The 22q13 region is represented with the genes (block: exon, line: intron, arrow: strand) and the regions corresponding to a higher than global prevalence for each feature (top). Regions significantly associated with absence of speech and ophthalmic features are also shown in more details (bottom). HI haploinsufficiency, pLI probability of loss-of-function intolerance. Color code similar to **a**

To map the genomic regions associated to PMS-related features, we measured the prevalence of ASD traits, absence of speech, ophthalmic features, seizures, gastroesophageal reflux, heart abnormalities or corpus callosum abnormalities in sliding windows of 1.5 Mb, each 50 kb in the 22q13 region (Fig. 3, Materials and Methods). Using this approach, we mapped candidate 22q13 regions conferring a high risk to display specific clinical features, which were statistically assessed for enrichment (odds ratio (OR) and *p*-value) and for statistical power to identify such a region given the sampling of the cohort (sampling *p*-value, Materials and Methods). We found regions with significantly higher risk for absence of speech, ophthalmic features and gastroesophageal reflux. To identify candidate genes within these regions, we mapped their corresponding haploinsufficiency ranks (HI) and probabilities to be intolerant to loss-of-function mutations (pLI) (Supplementary Fig. 3).

The prevalence for absence of speech was 46 ± 7% in the cohort, but increased to 80% for individuals who carry deletions including the genomic segment between position 42.6–46.3 Mb on chromosome 22 (enrichment OR = 13.8 [2.5–145.1], *P* = 0.0005 and sampling *P* = 0.003, Fig. 3). Thirty-eight protein-coding genes are located within this region, including *PACSIN2, MPPED1, SULT4A1* and *ATXN10* that are all expressed in the brain, are highly haploinsufficient and/or likely to be intolerant to loss-of-function mutations and therefore represent compelling candidates for increasing the risk of absence of speech in individuals with PMS.

The overall prevalence of ophthalmic features was 30%, but increased to 70% when the deletions include the 42.25–44.6 Mb region (enrichment OR = 10.3 [2–74.6], *P* = 0.002 and sampling *P* = 0.023, Fig. 3). Two genes are located at the boundary of this region and represent compelling candidates with low HI ranks and high pLIs: *TCF20* encodes a transcriptional co-activator previously associated with ASD^24^ and *SREBF2* encodes a transcription factor highly expressed in the brain.

The prevalence of gastroesophageal reflux was 19% in the cohort, increased to 70% when deletions included the 48.9–49.9 Mb region (enrichment OR = 12.3 [1.4–163.9], *P* = 0.009 and sampling *P* = 0.026). This region contains the *FAM19A5* gene, encoding a small secreted protein mainly expressed in the brain and potentially acting as a modulator of immune response in nervous cells.^25^

For the remaining clinical features associated with PMS (ASD traits, heart abnormalities, seizures and corpus callosum abnormalities), no genomic regions were significantly associated with increased risk and supported by both enrichment and sampling statistical measures, suggesting that other loci or environmental factors could act as modifiers modulating the presence or severity of these features in PMS. Of note, as previously noted in several publications, it may also be more difficult to evaluate ASD in patients with severe developmental, speech, and motor impairments, which are associated with larger deletions.

### Additional copy-number variants (CNVs) in patients with PMS

**Fig. 4:**
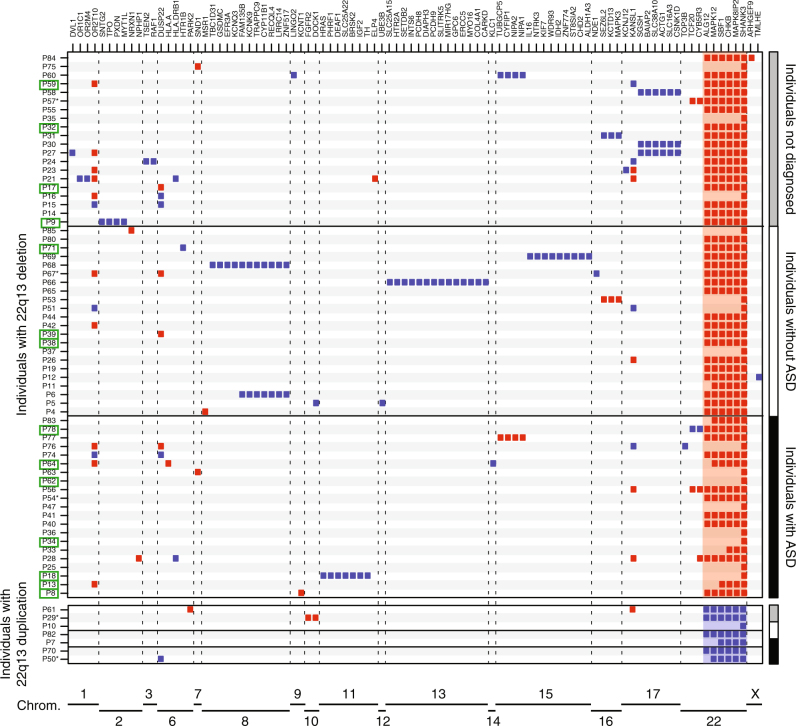
Additional CNVs identified in the cohort and including at least one NP-gene. Deletions (red) and duplications (blue) are represented for each patient and each NP-gene. Green squares indicate individuals with seizures. * indicate patients carrying mosaic CNVs

In order to test whether other loci could modulate the severity of the clinical features, we systematically identified all the CNVs carried by the patients and affecting the exons of 1,184 candidate neuropsychiatric risk genes (hereafter referred as NP-genes, Supplementary Table 4, Materials and Methods). Among the 63 patients carrying 22q13.3 deletions and tested using an array technology, 41 carried at least one CNV including at least one exon of a NP-gene (Fig. 4, Supplementary Fig. 4, Supplementary Table 5). Twenty-five patients had only one such CNV, nine had two, and seven had three or more. This burden of CNVs affecting NP-genes did not differ from the one measured in independent cohorts of patients with NDD and tested on similar array platforms (Supplementary Fig. 5). Despite a slightly higher number of CNVs in patients showing specific clinical features (absence of speech, seizures, heart abnormalities, corpus callosum abnormalities), we did not observe a statistically significant correlation between the burden of CNVs and the clinical features (Supplementary Fig. 6). Interestingly, we found five patients who carried CNVs recurrently associated with ASD (Supplementary Information). Patient P31 carried a 854 kb duplication at 16p11.2 inherited from a healthy mother, patient P53 carried a 494 kb deletion at 16p11.2 of unknown inheritance, patient P60 carried a 300 kb duplication at 15q11.2 of unknown inheritance, patient P67 carried a 2.7 Mb duplication at 16p12.3 of unknown inheritance, and patient P77 carried a 475 kb deletion at 15q11.2 inherited from a healthy father.

Out of the 14 patients carrying 22q13 deletions and having seizures, 4 were carrying an additional CNV covering a known risk gene for epilepsy: *KCNT1* (OMIM: 608167), *MYT1L* (OMIM: 613084), *DEAF1* (OMIM: 602635) and *SLC25A22* (OMIM: 609302) (Fig. 4, Supplementary Fig. 4). For example, patient P8, who was severely affected and unable to speak, had a ring chromosome 22 resulting in a terminal 22q13 deletion of 7.1 Mb and an additional 87 kb deletion at 9q34 including *KCNT1* that codes for a sodium-activated potassium channel, largely expressed in the nervous system and implicated in autosomal dominant forms of epilepsy.

### A multiplex family with an inherited *SHANK3* deletion

**Fig. 5:**
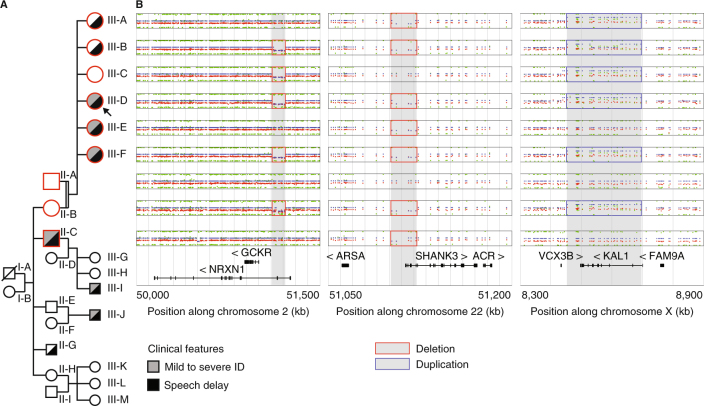
Multiplex family with inherited *SHANK3* and *NRXN1* deletions and *KAL1* duplication. **a** The nine studied individuals are displayed in the pedigree of the family (red border). **b** CNVs detected by OmniExpress Illumina arrays and including the *NRXN1, SHANK3* and *KAL1* genes (log2 scale)

To illustrate the genetic and clinical heterogeneity of patients carrying *SHANK3* deletions, we further investigated one multiplex family including one patient of this study. In this family, we identified one female patient (P85, subject III-D, Fig. 5) who carried a maternally inherited *SHANK3* deletion of 67 kb, removing exons 1–8 of the isoform A. In addition, she also carried a 104 kb deletion of *NRXN1* at 2p16.3 (exons 3–5 of the Alpha 2 isoform and exons 3–4 of the Alpha 1 isoform) and a 255 kb duplication on chromosome Xp22.31 including the Kallman syndrome gene *KAL1*. This proband was a member of a multiplex family that illustrates the heterogeneous clinical severity and the presence of multiple hits in the genome of the patients.

The proband was a 10-year-old female, fourth child (among six) of consanguineous parents (2nd degree). Birth and neonatal parameters were in the normal range. During infancy, the parents reported paucity of social interactions, but without stereotyped body movements. The patient exhibited delayed speech, with first words between 3 and 4 years old. At 8 years old, she was diagnosed with ID, and assessed for cognitive impairment (full scale IQ = 49, below the first percentile for her age). She did not meet criteria for ASD or any additional axis I psychiatric comorbidities. The patient presented with minor dysmorphic features including a curved forehead with a high implantation of her hair, a short and broad nose with flat nasal tip, long flat philtrum, a thick upper lip with a retrognathy. Concordant with her cognitive defects, she attended a special needs school and acquired basic writing and reading skills.

The father had no personal medical or psychiatric history and carried none of the three CNVs including *SHANK3*, *NRXN1* and *KAL1*. In contrast, the mother (subject II-A, Fig. 5) carried the two deletions including *SHANK3* and *NRXN1*, and the duplication including *KAL1*. She had mild learning difficulties related to a borderline intelligence quotient (IQ; non-verbal IQ = 75), but no history of speech delay. She could make sentences with more than three words at two-and-a-half years old. The clinical assessment of the mother revealed that she had no significant autistic symptoms, no axis I psychiatric comorbidities and no significant medical history. She shared similar signs of dysmorphism with her daughter.

We also investigated the sisters of the proband (subject III-A, III-B, III-C, III-E and III-F; Fig. 5, Supplementary Information). Only one girl (III-C) did not carry the *SHANK3* deletion. She had a full scale IQ in the normal range (FS-IQ = 92). She did not display any significant autistic symptoms (Social Responsiveness Scale total score <66 percentile) or any signs of developmental delay (specifically no significant delay in speech development). The four other girls presented either moderate speech delay with first sentences at 4 years old (III-A, III-B), or both speech disorder and mild to moderate ID (III-E, III-F). However, none of them displayed significant autistic symptoms, nor axis I psychiatric comorbidities and significant medical history. Finally, we investigated one of the maternal uncles (subject II-C, Fig. 5), who also carried the *SHANK3* deletion and presented mild ID associated with a severe language impairment. He currently has a job dedicated to persons with mental health problems.

Genetic testing showed that the five affected daughters and the maternal uncle carried the 22q13 deletion removing the first part of isoform A of *SHANK3*. In addition, subjects III-B, III-C, III-D, III-F and II-B also had the 2p16.3 deletion overlapping *NRXN1* (Fig. 5), and subjects III-A, III-B, III-C, III-D, III-F and II-B also had the Xp22.31 duplication overlapping *KAL1*. All affected subjects carried the partial *SHANK3* deletion suggesting its pathogenicity in the syndrome.

Finally, we investigated the potential impact of recessive mutations in all six daughters by locating runs of homozygosity (Supplementary Fig. 7). We identified chromosomal regions found specifically in the girls presenting speech delay, specifically in the girls presenting ID, and specifically in the girls not presenting ID. Interestingly, the only genomic region shared selectively by the three girls presenting ID included *DLGAP2*, an ASD-risk genes and a well established protein partner of SHANK3 at glutamatergic synapses.^26^

## Discussion

### Prevalence of PMS, ASD, seizures, and brain structural abnormalities

Our broad estimation of the prevalence of patients with PMS in patients with NDD is 0.27% (0.21–1.38%), which is similar to the ones reported previously (0.2–0.4%).^2^ We also observed a balanced sex ratio as previously reported.^1^ Regarding the clinical features (Supplementary Table 2, Supplementary Information), we confirmed previous reports: in most cases, patients with PMS received a diagnosis of NDD, ID and/or speech impairment.

In our cohort, 50% of the patients were reported with autistic traits. In the literature, the frequency of patients with PMS diagnosed with ASD varied significantly from one study to another mostly because of inconsistent methods of evaluation. In a report of eight patients, Philippe et al. (2008) did not diagnose ASD in any of the patient.^27^ In contrast, Phelan et al. (2012) diagnosed ASD in 17 out of 18 patients with PMS.^1^ Soorya et al. (2013) showed that 27 out of 32 patients with PMS (84%) have a diagnosis of ASD when standardized methods are used.^18^ Based on standardized interview with parents/caretakers, a recent study estimated that approximately 50% of PMS patients met criteria for ASD (21 patients out of 40).^28^ Similarly, the prevalence for seizures seems highly heterogeneous among studies since it ranges from 17 to 70% in multiple case series.^29^ In Kolevson et al. (2014), 25% of the patients from 13 independent studies (121/482) had seizures,^16^ a frequency very similar to this cohort (24%; 19/78) (Supplementary Information).

In the literature, brain structural abnormalities were reported in six studies, with a mean rate of 25% (58/233) of cases.^16^ Hypoplasia of the cerebellar vermis,^30–33^ thin corpus callosum,^18,27,30,34^ and abnormalities of white matter were previously reported. In our cohort, brain MRI data was available for 35 patients with 22q13 deletions and 23 had structural anomalies (65.7%), consistent with those already described in PMS (Supplementary Information).

### Impact of the 22q13.3 deletion size on the absence of speech

We observed that ASD was associated with smaller deleted segments, and could confirm that absence of speech was associated with large deletions.^17,20^ Similarly, Sarasua et al. (2014) reported that patients with an absence of speech had deletions in the 43.17–46.73 Mb interval.^17^ In our study, patients with breakpoint proximal to 46 Mb were more at risk for absence of speech than patients with smaller distal deletions (Figs. 2, 3). Within the 42.6–46.25 Mb interval, several genes are expressed in the brain and are compelling candidate for increasing the risk of absence of speech. *PACSIN2* (protein kinase C and casein kinase substrate in neurons 2) is a member of the pacsin-syndapin-FAP52 gene family and is upregulated upon neuronal differentiation. It has an essential role in the organization of clathrin-mediated membrane endocytosis in neurons.^35^ The function of *MPPED1* remains uncharacterized, but it codes for a metallophosphoesterase domain-containing protein highly expressed in the human fetal brain. *SULT4A1* codes for a protein of the sulfotransferase family involved in the metabolism of endogenous factors such as hormones, steroids, and monoamine neurotransmitters, as well as drugs and xenobiotics. *SULT4A1* is exclusively expressed in neural tissues, is highly conserved, and has been identified in all vertebrate studied so far. Interestingly, zebrafish carrying homozygous *SULT4A1* mutations exhibit excessively sedentary behavior during the day,^36^ suggesting a role of this gene in regulating behavior. The function of *ATXN10* remains largely unknown, but expanded ATTCT pentanucleotide repeats in intron 9 of the gene cause a rare form of spinocerebellar ataxia (SCA10) characterized by cerebellar ataxia and epilepsy.^37^

Interestingly, the region associated with absence of speech did not include *WNT7B*, a member of the WNT signaling molecule involved in the formation of the central nervous system vascular endothelium^38^ as well as in dendrite development.^39^ In contrast, this region includes the minimal interval of interstitial 22q13 deletions (not involving *SHANK3*, Supplementary Fig. 8) causing clinical features common to PMS^8^ and including *SULT4A1* and *PARVB*. *PARVB* is not highly expressed in the brain, but it codes for an actin-binding protein that interacts with ARHGEF6, a protein coded by an X-linked gene mutated in patients with ID.^40^

Only few studies investigated the prevalence of clinical features in the 22q13 region,^19,20^ but are missing standardized genetic and clinical data (Supplementary Fig. 8). Applying our framework on large cohorts of well-phenotyped individuals with genetic profiling based on homogeneous array technologies may lead to a more detailed map of the regions in 22q13 associated with specific clinical features of PMS.

### Multiple hits in patients with PMS

We also investigated the presence of multiple-hits in patients with PMS. Among the 63 patients with array results, 41 carried at least one additional CNV encompassing exonic sequences of one NP-gene. It is important to consider that a duplication of a gene might not be causative and therefore CNVs affecting NP-genes might not always be deleterious. Nevertheless, we found 16p11.2 CNVs (one deletion and one duplication) in two independent patients (P32 and P59), which are known to increase risk of NDD^41,42^ and are rare in the general population (0.03%).^2^ The finding of two cases with such CNVs is intriguing and raises question of the genetic architecture of PMS, and by extension on the cumulative genetic risk factors in NDD.^43^ In addition, we observed abnormal gene dosage of risk-genes for epilepsy such as *KCNT1*, *MYT1L*, *DEAF1* and *SLC25A22* in patients whom had lifetime history of seizures. However, at that stage, we cannot conclude on the causative effect of such CNVs since we also observed patients with CNVs affecting risk-genes for epilepsy (such as *KANSL1* (refs. 44, 45) or *NRXN1* (ref. 46)), and a lifetime absence of epilepsy.

### A multiplex family with an inherited *SHANK3* deletion

The clinical and genetic heterogeneity of the members of the multiplex family represent a proof-of-concept of the existence of multiple-hits in PMS. However, it remains difficult to identify the genes contributing to the severity of the symptoms. The 2p16.3 deletion including the *NRXN1* gene coexists in three patients with the 22q13 deletion, two with ID and speech delay (III-D and III-F) and one with only speech delay (III-B). This CNV could act as a modifier factor contributing at least in part to the clinical variability of the syndrome in this family. The unaffected girl (III-C) carried only the *NRXN1* deletion and the *KAL1* duplication, confirming that these CNVs display incomplete penetrance and/or variable expressivity. Accordingly, the chromosomal region including *KAL1* is highly polymorphic in the general population (Supplementary Fig. 9), hindering simple statistical association with PMS symptoms. In summary, multiple-hits exist in patients with PMS, but their impact on the severity of the symptoms remains to be determined in larger cohorts of well-phenotyped patients with more extensive genetic profiles using whole exome/genome sequencing.

## Conclusions

Our study confirms previous findings regarding the impact of the 22q13 deletion on several clinical features such as absence of speech and ASD. We also identified one mother without ID nor ASD carrying a *SHANK3* deletion, providing the proof of principle that some individuals could be resilient for such mutations. Larger cohorts of individuals carrying 22q13 deletions (including interstitial deletions not affecting *SHANK3*) with in-depth phenotyping and whole genome sequencing data should allow us to identify modifier genes. Such genes and pathways would contribute to our understanding of the etiology of PMS and could represent new relevant drug targets.

## Materials and methods

### Population

Eighty-five patients (39 males, 44 females and two fetuses) carrying a genomic rearrangement at the 22q13 region encompassing *SHANK3* were recruited through a French national network of cytogeneticists from 15 centers (ACHROPUCE network). For seven centers, the total number of microarray analyses performed for ID, congenital anomalies or ASD was available to establish a broad estimation of the prevalence of *SHANK3* deletions (Supplementary Table 1). Detailed clinical information was collected for all subjects based on medical records, including perinatal events, growth parameters at birth and during early life of developmental, cognitive and functional development, congenital anomalies, dysmorphic features and main somatic comorbidities (Fig. 1, Supplementary Table 2, Supplementary Information). Results from electroencephalograms and brain MRI were also gathered for 21 and 35 individuals, respectively. We used the Diagnostic and Statistical Manual of Mental Disorder Fifth edition criteria for ASD diagnostic, and standardized evaluations including the Autism Diagnostic Interview Revised^47^ and the Autism Diagnostic Observation Schedule.^48^ The cognitive level was also measured using Raven’s Progressive Matrices for non-verbal IQ and the Peabody picture vocabulary test for verbal IQ. Institutional Review Board approval was not required for this study. The methods were performed in accordance with relevant guidelines and regulations. For each patient, a parent or legal guardian provided written informed consent and retained the right to oppose the use of the data at any time. For the two fetuses, after the detection of a major fetal malformation revealed by echography, the parents consented for sampling the fetuses for genetic examination. The tissue sample was obtained by a trophoblast biopsy. Informed consent was obtained for each patient to publish the images in Supplementary Fig. 1.

### Identification of genomic rearrangements

Genomic data included information on the type of genomic rearrangement at the 22q13 region, the methods of detection and confirmation, the molecular coordinates and the inheritance status (Supplementary Table 3). We considered the size and breakpoints of the 22q13 rearrangement only for 74 patients studied using microarray technologies (Supplementary Fig. 10, Supplementary Table 3).

Quality controls were performed locally in each genetic laboratory (Supplementary Table 3). Genomic coordinates of each CNV are given according to the hg19 version of the human reference genome. All 22q13 rearrangements were validated in the corresponding clinical center using independent molecular technologies such as FISH, qPCR or MLPA. Inheritance of the 22q13 rearrangement was also determined in the corresponding laboratories.

We mapped all CNVs in the genomes of the patients, and focused on a list of 1,184 candidate genes for neuropsychiatry (NP-genes), which included Class I-III genes,^49^ TADA-65 genes,^50^ the genes from the SFARI database (release from the 9^th^ November 2015; https://gene.sfari.org), and the developmental brain disorder genes (Supplementary Tables 4 and 5).^51^ Four patients (P20, P22, P79 and P81) were not included in the analysis since their arrays did not pass the quality control for a whole-genome analysis. In 14 cases with unbalanced reciprocal translocations (Supplementary Table 3), we considered the other chromosomal unbalanced segments as additional CNVs. The burden of CNVs in patients with PMS was compared to independent cohorts of patients with NDD (Supplementary Fig. 5).

### Computational and statistical analyses

Most analyses were performed on genetic and clinical data from 74 patients with array data (67 with a 22q13 deletion, 7 with a duplication), excluding 11 patients for whom only standard cytogenetic data were available (Supplementary Table 3).

Ward’s hierarchical agglomerative clustering analysis was performed using JMP Pro 10.0.2 (http://www.jmp.com) on 37 individuals for whom data on ASD, speech, seizures and deletion size were available (Supplementary Table 2). For 12 patients with SNP data (six patients used in the clustering analysis), we could estimate the genetic ancestry using genome-wide pairwise identity-by-state (IBS, Supplementary Fig. 11). Some of the patients clustering together were not sharing the same ethnicity. The IBS analysis was performed using PLINK and ~20,000 SNPs overlapping from genotyping data of HapMap3 populations (Illumina Human1M and Affymetrix SNP 6.0) and the 12 patients of our cohort (four Illumina Infinium OmniExpress-24 and eight Illumina HumanCytoSNP-12). All SNPs with Hardy–Weinberg equilibrium *p*-values below 0.001 were removed.

For the mapping of the 22q13 region, the prevalence of each feature was measured each 50 kb in sliding windows of 1.5 Mb. For each window, we calculated the percentage of individuals carrying a CNV starting in the corresponding interval and showing ASD, absence of speech, ophthalmic features, seizures, gastroesophageal reflux, heart abnormalities or corpus callosum abnormalities. Then, for each feature, we identified the interval defined by the minimal and maximal positions at which the prevalence was higher than the overall prevalence of the feature in the cohort. Finally, the association of the region with the feature was measured by a Fisher’s exact test for the enrichment OR and *p*-value of individuals having the feature and carrying a CNV starting in the region compared to the rest of the 22q13 region. To measure the probability to find such regions given the clinical and genetic sampling of the cohort, we developed a bootstrap-based measure of the statistical power. For each feature, we re-shuffled 1,000 times the feature presence/absence of the individuals, and reran our analysis of prevalence. We counted the number of times the enrichment OR was higher than the one observed with the original data, and used it as an empirical *p*-value (hereafter referred to as sampling *p*-value).

For all genes in the 22q13 region, we mapped the corresponding HI ranks (downloaded from DECIPHER database on May 2017)^52^ and probability of intolerance to loss-of-function mutations (pLI, downloaded from the Broad Institute website on March 2016).^53^

We searched for runs of homozygosity in the multiplex family using PLINK and ~700,000 SNPs present in all nine individuals and two additional saliva samples (individuals III-B and III-E). All SNPs with Hardy–Weinberg equilibrium *p*-values below 0.001 were removed.

### Data availability

The datasets generated during and/or analyzed during the current study are available in Supplementary Information.

## Electronic supplementary material

Supplementary Information

Supplementary Figure 1

Supplementary Figure 2

Supplementary Figure 3

Supplementary Figure 4

Supplementary Figure 5

Supplementary Figure 6

Supplementary Figure 7

Supplementary Figure 8

Supplementary Figure 9

Supplementary Figure 10

Supplementary Figure 11

Supplementary Table 1

Supplementary Table 2

Supplementary Table 3

Supplementary Table 4

Supplementary Table 5

Supplementary Table 6

## Electronic supplementary material

**Supplementary information** accompanies the paper on the *npj Genomic Medicine* website (10.1038/s41525-017-0035-2).
