# Author Correction: A framework to identify contributing genes in patients with Phelan-McDermid syndrome

**DOI:** 10.1038/s41525-019-0090-y

**Published:** 2019-07-01

**Authors:** Anne-Claude Tabet, Thomas Rolland, Marie Ducloy, Jonathan Lévy, Julien Buratti, Alexandre Mathieu, Damien Haye, Laurence Perrin, Céline Dupont, Sandrine Passemard, Yline Capri, Alain Verloes, Séverine Drunat, Boris Keren, Cyril Mignot, Isabelle Marey, Aurélia Jacquette, Sandra Whalen, Eva Pipiras, Brigitte Benzacken, Sandra Chantot-Bastaraud, Alexandra Afenjar, Delphine Héron, Cédric Le Caignec, Claire Beneteau, Olivier Pichon, Bertrand Isidor, Albert David, Laila El Khattabi, Stephan Kemeny, Laetitia Gouas, Philippe Vago, Anne-Laure Mosca-Boidron, Laurence Faivre, Chantal Missirian, Nicole Philip, Damien Sanlaville, Patrick Edery, Véronique Satre, Charles Coutton, Françoise Devillard, Klaus Dieterich, Marie-Laure Vuillaume, Caroline Rooryck, Didier Lacombe, Lucile Pinson, Vincent Gatinois, Jacques Puechberty, Jean Chiesa, James Lespinasse, Christèle Dubourg, Chloé Quelin, Mélanie Fradin, Hubert Journel, Annick Toutain, Dominique Martin, Abdelamdjid Benmansour, Claire S. Leblond, Roberto Toro, Frédérique Amsellem, Richard Delorme, Thomas Bourgeron

**Affiliations:** 1 Genetics Department, Robert Debré Hospital, APHP, Paris, France; 2 Human Genetics and Cognitive Functions, Institut Pasteur, Paris, France; 3 CNRS UMR 3571 Genes, Synapses and Cognition, Institut Pasteur, Paris, France; 4 Sorbonne Paris Cité, Human Genetics and Cognitive Functions, Université Paris Diderot, Paris, France; 5 Cytogenetics Unit, Pitié Salpetrière Hospital, APHP, Paris, France; 6 Neurogenetics Unit, Pitié Salpetrière Hospital, APHP, Paris, France; 7 Clinical Genetics Unit, Pitié Salpetrière Hospital, APHP, Paris, France; 8 Cytogenetics Unit, Jean Verdier Hospital, APHP, Bondy, France; 9 Cytogenetics Unit, Trousseau Hospital, APHP, Paris, France; 10 Clinical Genetics Unit, Trousseau Hospital, APHP, Paris, France; 11 Clinical Genetics Unit, Nantes Hospital, Nantes, France; 12 Cytogenetics Unit, Cochin Hospital, APHP, Paris, France; 13 Genetics Unit, CHU Estaing, Clermont-Ferrand, France; 14 Cytogenetics Unit, Dijon Hospital, Dijon, France; 15 Clinical Genetics Unit, Dijon Hospital, Dijon, France; 16 Genetics Unit, La Timone Hospital, Marseille, France; 17 Cytogenetics Unit, Lyon Civil Hospital, Lyon, France; 18 Clinical Genetics Unit, Lyon Civil Hospital, Lyon, France; 19 Cytogenetics Unit, Grenoble Hospital, Grenoble, France; 20 Clinical Genetics Unit, Grenoble Hospital, Grenoble, France; 21 Genetics Unit, Bordeaux Hospital, Bordeaux, France; 22 Genetics Unit, Montpellier Hospital, Montpellier, France; 23 Genetics Unit, CHRU Nimes, Nimes, France; 24 Cytogenetics Unit, Chambéry-Hôtel-Dieu Hospital, Chambéry, France; 25 Genetics Unit, CHU Rennes, Rennes, France; 26 Genetics Unit, Chubert Hospital, Vannes, France; 27 Genetics Unit, Bretonneau Hospital, Tours, France; 28 Genetics Unit, CH Le Mans, Le Mans, France; 29 Department of Child and Adolescent Psychiatry, Robert Debré Hospital, APHP, Paris, France

**Keywords:** Clinical genetics, Neurodevelopmental disorders

**Correction to:** *npj Genomic Medicine* 10.1038/s41525-017-0035-2, published online 23 October 2017

In the original published version of the Article, the ‘Population’ section included the statement that “the local Institutional Review Boards (IRB) at each clinical center approved the study. The methods were performed in accordance with relevant guidelines and regulations and approved by the Institut Pasteur IRB”. However, IRB approval was not granted because it was not necessary for this study. French regulations do not require IRB approval for studies which involve the reutilization of data provided that subjects provide written informed consent and retain the right to oppose the use of data at any time. All patients provided written informed consent for this study.

The statement was therefore updated to “Institutional Review Board approval was not required for this study. The methods were performed in accordance with relevant guidelines and regulations. For each patient, a parent or legal guardian provided written informed consent and retained the right to oppose the use of the data at any time” in the PDF and HTML versions of the Article.

