# Supplementary material for: A framework to identify contributing genes in patients with Phelan-McDermid syndrome
Source: NPJ Genom Med. 2017 Oct 23;2:32. doi: 10.1038/s41525-017-0035-2 (PMC5677962; doi:10.1038/s41525-017-0035-2)
Supplement: Supplementary file 1 — Supplementary Information [file 41525_2017_35_MOESM1_ESM.docx]

**TABLE OF CONTENT**

| **Supplementary Figure Legends………………………………………………….** | **2** |
| --- | --- |
| **Supplementary Table Legends…………………………………………………...** | **4** |
| **Clinical Description of the Patients……………………………………………...** | **5** |
| **Case Reports of Patients with a Second Hit involved in NDD………………** | **10** |
| **Supplementary References……………………………………………………….** | **22** |

**SUPPLEMENTARY FIGURE LEGENDS**

**Supplementary Figure 1. IRM images.** Images are shown for patients with a normal (**A**-**B**) or abnormal (**C**-**D**) corpus callosum, indicated by red squares. Informed consent was obtained for each patient to publish the images.

**Supplementary Figure 2. 22q13 CNVs in the 85 studied patients.** Deletions (red) and duplications (blue) are represented along the 22q13 locus. Clinical features of each patient are indicated on the right part of the figure (black: has the feature, white: doesn’t have the feature, grey: not tested). * indicate patients carrying mosaic CNVs.

**Supplementary Figure 3. Haploinsufficiency and pLI score distribution in 22q13 regions of high prevalence.** (**A**) The HI and pLI scores are compared between the genes within the 22q13 region (“Within”) and the rest of the genome (“Rest”). P-values were measured by two-sided Wilcoxon rank sum tests. (**B**) For each feature, the HI and pLI scores are compared between the genes within the high prevalence region in 22q13 (“Yes”), the other genes in the 22q13 region (“No”), and the rest of the genome (“Rest”). P-values were measured by two-sided Wilcoxon rank sum tests.

**Supplementary Figure 4. Additional CNVs including NP-genes.** (**A**) Patients with 22q13 deletions. (**B**) Patients with 22q13 duplications. Only patients with at least one other CNV including a NP-gene are represented, and only CNVs that are not including *SHANK3* are represented. * indicate patients carrying mosaic CNVs.

**Supplementary Figure 5. Burden of CNVs in the cohort.** The number of CNVs per individual in our cohort (22q13 CNV) is compared for Agilent 180K and 60K as well as Illumina Cyto-SNP12 array technologies to the ones measured in other cohorts of NDD individuals. For each comparison, individuals are divided between those carrying a CNV recurrently associated to NDD and those who don’t. Numbers of individuals are given for all identified CNVs (left) or for CNVs including at least one NP-gene (right), and for CNVs of size < 250 kb (top) or >= 250 kb (bottom).

**Supplementary Figure 6. CNV load in individuals with or without clinical features.** For each feature, the fraction of individuals with the corresponding number of CNVs involving a NP-gene and having (grey) or not having (white) the feature over those with the corresponding number of CNVs is shown. Enrichment odds ratios and 95% confidence intervals were calculated using Fisher exact tests.

**Supplementary Figure 7. Runs of homozygosity in the daughters of the multiplex family.** All ROHs are represented in the circos plot (top), and specific regions are represented with the corresponding genes (bottom).

**Supplementary Figure 8. Meta-analysis of the regional prevalence in the 22q13 region.** Only significantly associated regions are shown.

**Supplementary Figure 9. Screenshot of the region surrounding *KAL1* in DGV.**

**Supplementary Figure 10. CNV identification platforms.** For each array technology, the number of patients tested is given.

**Supplementary Figure 11. Multi-Dimensional Scale Analysis of the genetic distance between individuals of HapMap3 populations and our cohort.** Each dot represents an individual and the distance between two dots corresponds to genetic distance based on pairwise identity by state calculations.

**SUPPLEMENTARY TABLE LEGENDS**

**Supplementary Table 1. Prevalence.**

**Supplementary Table 2. Clinical data.**

**Supplementary Table 3. Genetic data.**

**Supplementary Table 4. NP-genes.**

**Supplementary Table 5. CNVs.**

**Supplementary Table 6. Birth parameters.**

**CLINICAL DESCRIPTION OF THE PATIENTS**

**Clinical features associated with PMS**

|  | Patients with 22q13 deletions |
| --- | --- |
| **Dysmorphic features** |  |
| Shape face anomalies | 19% (14/71) |
| Hypertelorism | 9% (6/71) |
| Wide nasal bridge | 6% (4/71) |
| Bulbous nose | 16% (11/71) |
| Ear anomalies | 22% (16/71) |
| Philtrum anomaly | 14% (10/71) |
| Micrognathia/retrognathia | 11% (8/71) |
| Dental anomalies | 10% (7/71) |
| Fifth finger clinodactyly | 6% (4/71) |
| Large fleshy hands | 12% (9/71) |
| Hypoplastic/dysplastic nails | 6% (4/71) |
| Syndactyly (2/3 of toes) | 11% (8/71) |
| **Neurodevelopmental disorder** | |
| Intellectual disability | 100% (66/66) |
| Speech delay | 52% (35/67) |
| Totally absent speech | 48% (32/67) |
| Autistic traits | 47% (28/60) |
| Aggressive behavior | 14% (9/63) |
| Anxiety | 4% (3/63) |
| Self-injury | 9% (6/63) |
| Hyperactivity | 12% (8/63) |
| Inappropriate cry | 4% (3/63) |
| **Other medical conditions** |  |
| Hypotonia | n/a |
| Seizures | 24% (19/78) |
| Heart abnormalities | 6% (5/74) |
| Ophthalmic features | 24% (19/78) |
| Gastroesophageal reflux | 16% (10/62) |
| Constipation/diarrhea | 10% (6/62) |
| Microcephaly | 7% (4/59) |
| Macrocephaly | 2% (1/59) |
| Abnormal onset of puberty | 7% (5/65) |
| Renal anomalies | 10% (7/65) |
| Short stature | 3% (2/62) |
| Tall stature | 5% (3/62) |
| Abnormal EEG | 30% (5/21) |
| Brain MRI anomalies | 65% (23/35) |

**Patients with 22q13 deletions**

**Pregnancy and early development.** During pregnancy, no specific events were reported except for 4/62 subjects with significant intrauterine growth retardation. Delivery was in the normal range for most patients of the cohort except for four cases with prematurity. Life and growth parameters at birth were also in the normal range (Table S6). Feeding difficulties were observed very early in 17 cases (21%). Hypotonia at birth and motor development delay were observed, but with variable severity: some patients acquired the ability to walk in the normal range of age, while some were unable to walk (P56 was the eldest one of the cohort at 19 years old, and has never been able to walk). The cohort also includes two fetuses (P27 and P30) that resulted from termination of pregnancy regarding severe fetal malformations: P27 presented with left diaphragmatic hernia, hydronephrosis, bicornuate uterus and dysgenetic sex disorder, and P30 had a complex heart malformation.

**Age at diagnosis.** Mean diagnosis age was 60.33 months for boys and 118.02 months for girls.

**Dysmorphic features.** Signs of dysmorphism were similar to those reported in the literature in patients with PMS.^1^ Additionally, shape face anomalies were reported in 14 cases, large, short and flappy hands in nine cases and dental anomalies in seven cases (Table S2).

**ID, ASD and associated psychiatric conditions.** Mild to profound ID was observed for all 66 patients for whom the information was available. Absence of speech was observed in half of the 60 patients for whom the information was available (including three that displayed loss of previously acquired words at early stage), the remaining patients displaying mild to severe speech difficulties. Half of the patients (28/63) received a diagnosis of ASD/Pervasive Developmental Disorder not otherwise specified (PDD-nos) based on the DSM-IV TR and clinical expertise. But only three of them had diagnosis using standardized assessment (ADI-R and ADOS). Additional psychiatric comorbidities were observed, including aggressive behaviors (14%, n=9/63), self-injurious behaviors (9%, n=6/63) and symptoms of attention deficit/hyperactive disorder (12%, n=8/63), anxiety disorders (4%, n=3/63) or bipolar disorder (1,5%, n=1/63).

**Somatic comorbidities**. Medical records reported that 24% of patients (n=19/78) displayed a personal history of seizures, 24% (n=19/78) of ophthalmic disorders (mostly refraction anomalies), 20% (n=13/62) of gastro-intestinal disorders (including gastroesophageal reflux in ten cases, merycism and chronic constipation), and 6% (n=5/74) of heart defects (four patients and one out of the two fetuses). Other medical conditions included abnormal growth stature (7%, n=5/65), abnormal onset of puberty (7%, n=5/65), pulmonary diseases (12%, n=8/65) (including recurrent upper respiratory tract infections and asthma), renal malformations (10%, n=7/65), skeletal abnormalities (15%, n=10/65), abnormal head circumference (8%, n=5/59) (four with microcephaly and one with macrocephaly) and deafness in two subjects (Table S2).

**Late onset cognitive skills regression.** Among the four cases for which long-term follow-up was available, three displayed late cognitive skills regression when they were over 15 years old. At 22 years old, P3 had a severe period of hyperactivity with lack of sleep that resulted in her exclusion from the educational structure for one month. After this period, she exhibited regression of learning abilities, specifically for speech. At 17 years old, despite anti-epileptic drugs, P9 suffered from an acute severe epileptic episode leading to loss of visual acuity and locomotion. At 20 years old, P83 also presented with learning regression associated with an increase of social communication deficit and stereotyped behaviors.

**Brain MRI and EEG explorations.** Among the 35 patients who had a structural brain MRI, 23 (65%) displayed brain structure anomalies. We observed corpus callosum defects (agenesis, thin or simplified) (43%, n=10), ventriculomegaly (26%, n=6) and decreased white matter with enlarged ventricles (39%, n=9). Two patients with microcephaly showed a thin corpus callosum (P26 and P65) and one had a thin brain stem (P26). One patient (P60) exhibited a Dandy Walker malformation (with a cerebellar vermis atrophy). Among 21 patients with EEG raw data, five showed minor EEG abnormalities but three of them had intercritic paroxysmal abnormalities.

**Patients with 22q13.3 duplications**

Our cohort included seven patients (five girls and two boys) with a 22q13 duplication. Birth parameters were unremarkable except for weight, which was decreased to an average of 2,367 g (under the 3^rd^ percentile) for boys and 2,722 g (at the 3^rd^ percentile) for girls (Table S6). Neurologic examination revealed axial hypotonia in one female (P29). We also reported autistic symptoms (n=3), ID (n=5), language delay (n=2) and absence of speech (n=4). One patient had significant psychiatric or cognitive symptoms (P7). Other medical features included chronic constipation/diarrhea, deafness, growth retardation, ophthalmic features and heart defect and obesity (Table S2). None of the patients suffered from seizures, gastroesophageal reflux and abnormal head circumference. Brain MRI was normal in two patients and showed enlarged Virchow-Robin spaces in one single case. EEG was performed for only one patient, and showed spike waves without epileptic activity.

**CASE REPORTS OF PATIENTS WITH A SECOND HIT INVOLVED IN NDD**

**Patient P31**

Center: Nantes

Technology: Agilent 60K ISCA (Array CGH)

First hit: de novo ring chromosome 22 resulting in 22q13 deletion (47,909,796-51,178,405), size: 3,268,609 pb

Second hit: 16p11.2 duplication (29,477,859-30,332,722), inherited from a healthy mother


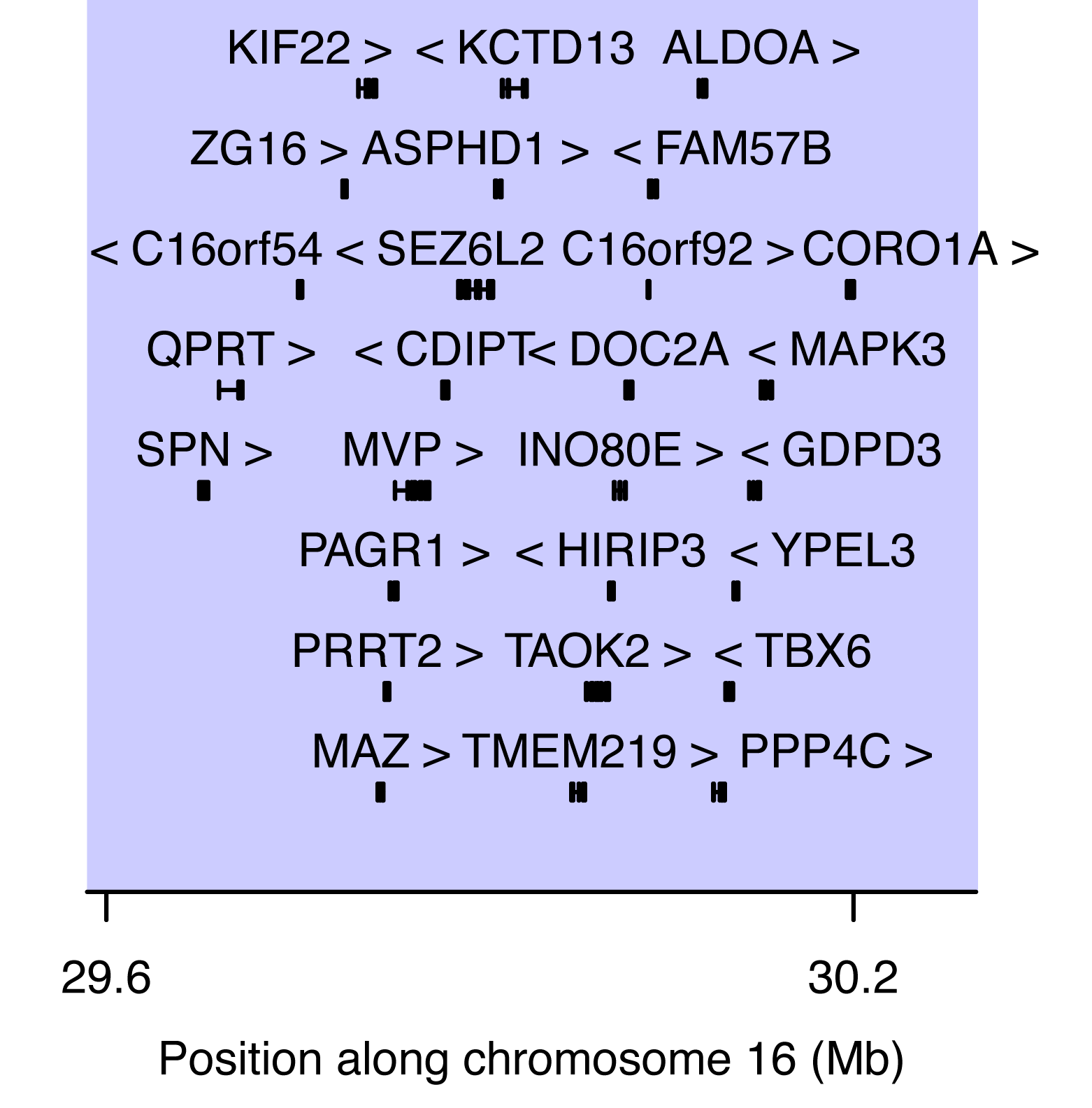


Snapshot of the 16p11.2 locus of P31 (signal from Agilent 60K ISCA CGH array) and genes affected by the duplication

Case report: The patient was a two-year-old boy. At the time of inclusion, he displayed minor dysmorphic features including a retrognathism. A severe hypotonia was present at birth, leading to feeding difficulties. He was unable to walk without assistance. At the cognitive level, he had a profound ID with totally absent speech. He also presented a supra aortic stenosis, but no other significant somatic comorbidity. Results from EEG and brain MRI were not available.

Neuropsychiatric impact of the 16p11.2 variation: The neuropsychiatric impact of the 16p11.2 duplication is discussed together with the 16p11.2 deletion carried by the next patient.

**Patient P53**

Center: Clermont-Ferrand

Technology: Agilent 180K (array CGH with Dye Swap revelation)

First hit: 22q13 deletion (51,116,107-51,170,223) of unknown inheritance, size: 54,116 bp

Second hit: 16p11.2 deletion (29,685,420-30,179,642) of unknown inheritance.

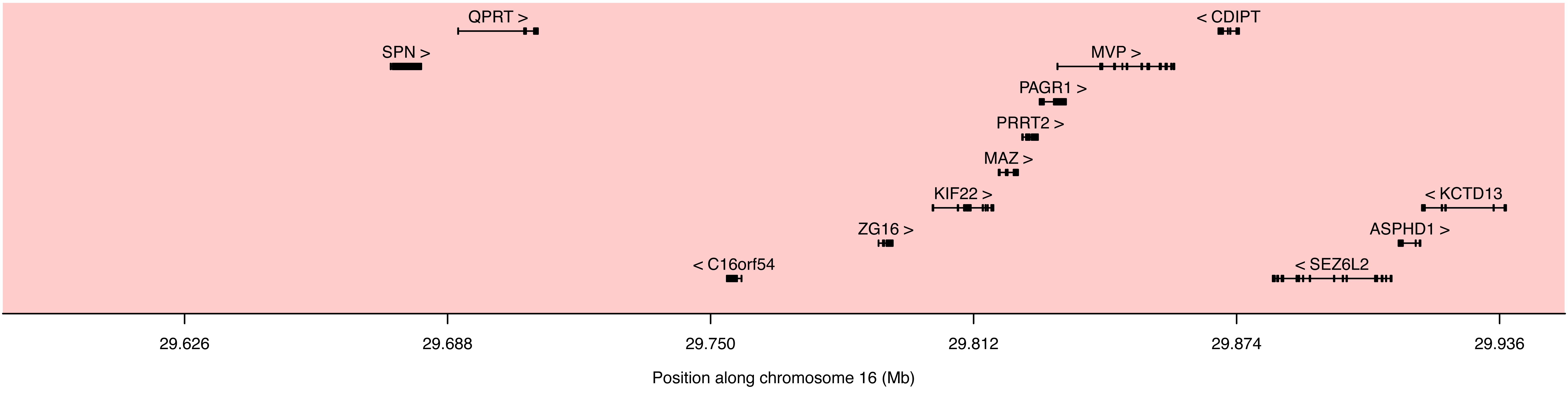


Snapshot of the 16p11.2 locus of P53 (signal from Agilent 180K CGH array and Dye Swap) and genes affected by the deletion

Case report: The patient was a eight-year-old boy with a global developmental delay. However, he had a borderline IQ and received a classical education supported by an individual scholar assistant. He had verbal communication but with a spoken language disorder. He did not display any autistic symptom and no major somatic comorbidity.

Neuropsychiatric impact of the 16p11.2 variation: The 16p11.2 deletion (a deletion of 494kb in this patient) represents one of the most common risk factor for neurodevelopmental disorders, accounting for example for 1% of all ASD cases.^2,3^ Deletions at this locus appear to be more penetrant than duplications. Patients carrying a 16p11.2 deletion have increased risk for macrocephaly and early onset obesity, while patients carrying the reciprocal duplication have higher risk of microcephaly and underweight.^4,5^

**Patient P60**

Center: Dijon

Technology: Agilent 180K (Array CGH)

First hit: de novo 22q13 deletion (46,244,344-49,525,271), size: 3,280,927 pb

Second hit: 15q11.2 duplication (22,784,323-23,085,237) of unknown inheritance

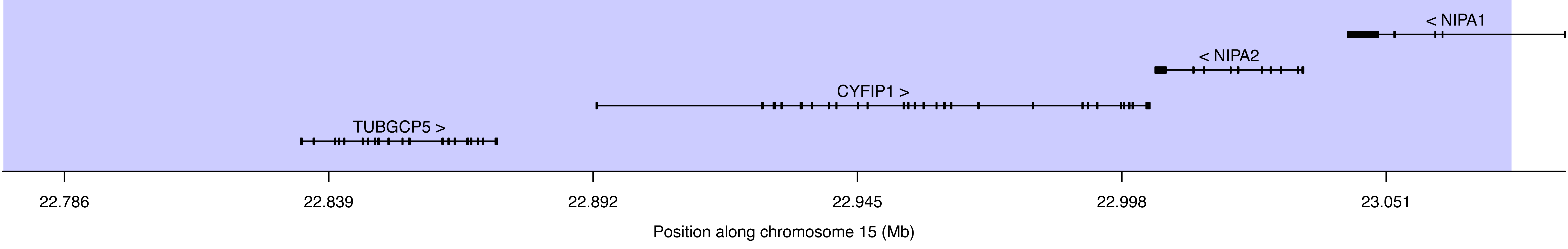


Snapshot of the 15p11.2 locus of P60 (signal from Agilent 180K CGH array) and genes affected by the duplication

Case report: The patient was an 18-month-old girl, presenting with unmotivated cry, poor social communication and interaction, poor eye contact, motor stereotypies and flapping. She did not speak at all. Her clinical exam revealed a microcephaly. She also suffered from gastroesophageal reflux.

Neuropsychiatric impact of the 15q11.2 duplication: The 15q11.2 duplication carried by the patient is reciprocal of the deletion discussed below (P77). Whether this duplication is also a risk factor for NDD is still controversial. Nevertheless, based on 1,654 children with various neurological disorders, a study showed that 12 patients carried a 15q11.2 duplication (0.7%), of whom 80% had developmental delay or ID.^6^

**Patient P67**

Center: Lyon

Technology: Agilent 180K (Array CGH with Dye Swap revelation)

First hit: de novo mosaic 22q13 deletion (43,057,749-51,219,150), size: 8,161,401 bp

Second hit: 16p12.3p13.1 duplication (15,492,117-18,162,308) of unknown inheritance

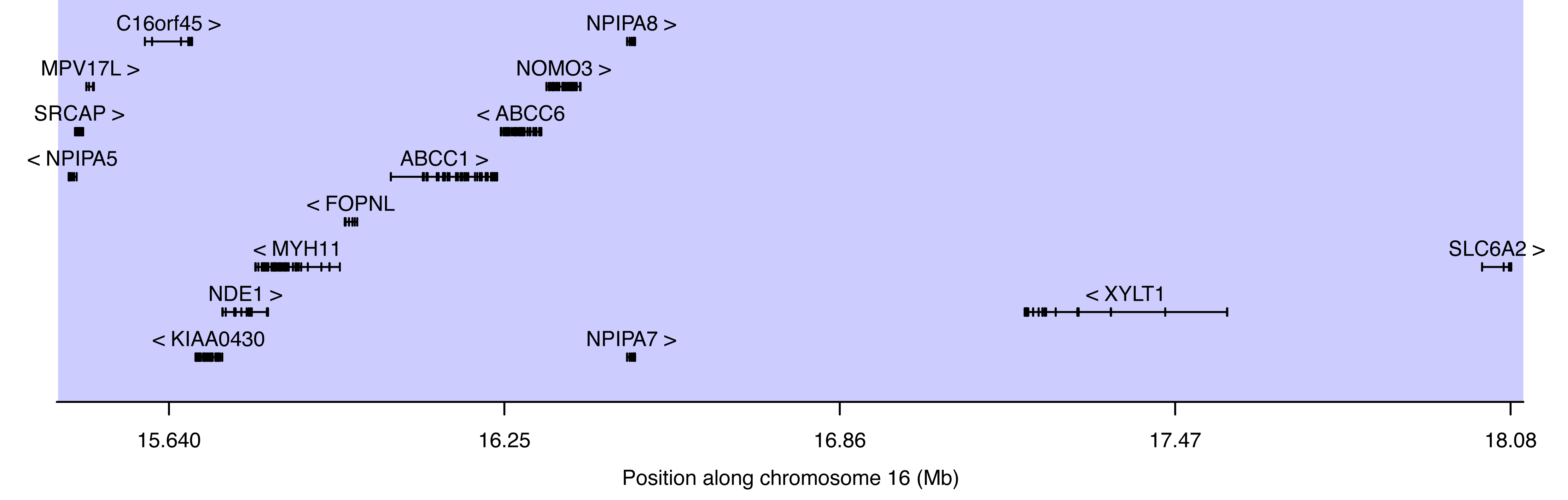


Snapshot of the 16p12.3p13.1 of P67 (signal from Agilent 180K CGH array and Dye Swap) and genes affected by the duplication

Case report: The patient was a 12-year-old boy. Main motor milestones were in the normal range. However, language was delayed and was not functional at the time of inclusion. He presented minor signs of dysmorphism, including asymmetric eyebrows and left ptosis. The neurologic exam was typical, except for an axial hypertonia and increased pain sensitivity. The EEG was normal.

Neuropsychiatric impact of the 16p12.3p13.1 duplication: The pathogenic CNVs at 16p13.1 were first described in severe autistic patients and unrelated patients with ID,^7^ then in patients with neurodevelopmental disorders or schizophrenia^8–10^ where males could be more affected than females.^10^ This CNV has also been repeatedly identified as inherited from mildly or unaffected parents, concordant with incomplete penetrance.

**Patient P77**

Center: Robert Debré

Technology: OmniExpress Illumina (SNP array)

First hit: de novo 22q13 deletion (47,648,233-51,211,392), size: 3,563,159 bp

Second hit: 15q11.2 deletion (22,750,305-23,226,254) inherited from a healthy father

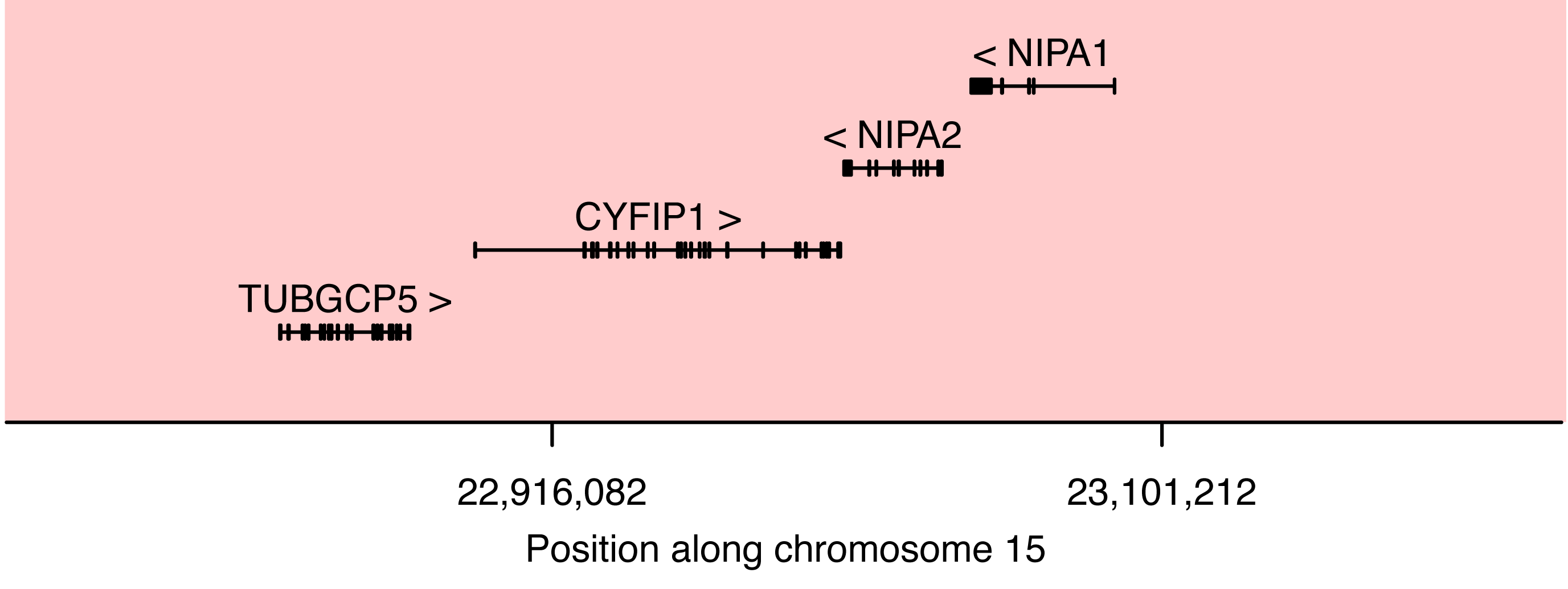


Snapshot of the 15q11.2 locus of P77 (signal from Illumina OmniExpress SNP array) and genes affected by the deletion

Case report: The patient was a 21-month-old girl. She presented autism features, ID, and had no verbal communication. Her clinical exam revealed hypertonia. Interestingly, a paternal aunt also had ID, but was not available for testing.

Neuropsychiatric impact of the 15q11.2 deletion: The 15q11.2 deletion between BP1 and BP2 has been recently reported. The 15q11.2 BP1-BP2 locus is approximately 500 kb long and involves four highly conserved and biallelically expressed genes (*NIPA1, NIPA2, TUBGCP5* and *CYFIP1*). In a cohort of 52 patients, abnormal phenotypes included mild to moderate developmental delay in 68.3% of the cases, speech impairment in 85.4 % and behavioural anomalies including ASD in 63.4%; furthermore, the deletion was inherited in most cases (81.2 %).^11^ In large studies, the prevalence of the deletion varies from 0.57%^12^ to 1.27%,^6^ a four-fold increase compared to controls.^13^ In a clinical review of 200 patients, developmental and speech delay were present in 73% and 67% of the cases, respectively, while general behavioural problems were observed in 55% of the cases, including ASD in 27% and ADHD in 35% of the cases.^13^ In a large sample of 2,525 families with autism, the deletion had a modest effect.^14^ In addition, maternally inherited deletions could be more penetrant in male carriers.^14^ Using iPSC-derived neuronal cells, Das et al. showed that the deletion was associated with decreased expression of the four genes and alteration of the morphology of dendritic spine.^15^

Therefore, this CNV should probably be interpreted as a risk factor for neurodevelopmental disorder. In our patient, the co-existence of both 22q13.3 and 15q11.2 deletions could be involved in the precocity and the severity of the phenotype.

**Patient P85**

Center: Robert Debré

Technology: OmniExpress Illumina (SNP array)

First hit: 22q13 deletion (51,072,161-51,139,178), size: 67,017 bp

Second hit: 2p16.3 deletion (51,108,133-51,212,752) and Xp22.31 duplication (8,439,382-8,694,839) both inherited from mildly affected mother (Figure 5)

Case report: The proband (subject III-D, Figure 5) was a 10-year-old female, fourth child (among six) of consanguineous parents (2nd degree). After a normal pregnancy and delivery (39 weeks of gestation), birth weight of the patient was 2990 g (10-50th percentile), length 50 cm (50-90th percentile), and occipito-frontal head circumference (OFC) 34 cm (50th percentile). APGAR scores were 10 at one and five minutes after birth. Despite normal development during infancy, including walking at 16 months, the parents reported paucity of social interactions, but without stereotyped body movements. The patient exhibited mild delayed speech, with first words between three and four years of age. Referred to a psychiatric unit at the age of eight years old, she was diagnosed clinically with ID, based on DSM-5. The assessment of her cognitive performance with the Wechsler Scale-children version, fourth edition, indicated that her IQ was below the first percentile for her age (full scale IQ: 49). She did not met criteria for ASD according to the ADI-R and the ADOS-2, neither for any additional axis I psychiatric comorbidities, which were systematically screened with the Kiddie-SADS. The patient's height, weight and OFC were all in the normal range, but she presented with minor dysmorphic features (not typical of PMS) including a curved forehead with a high implantation of her hair, a short and broad nose with flat nasal tip, long flat philtrum, a thick upper lip with a retrognathy. The neurological exam was also normal. Despite her cognitive defects, she attended a special needs school and acquired basic writing and reading skills.

The father, of North African origin, had no personal medical or psychiatric history. None of his three brothers and seven sisters displayed a history of NDD. In contrast, the mother (subject IIA, Figure 5), 41 years old, had mild learning difficulties related to a borderline IQ (Raven’s progressive Matrice: 5th percentile, Non-verbal IQ: 75), but she self-reported no history of speech delay. Similarly to her daughter, she carried the three CNVs involving *SHANK3*, *NRXN1* and *KAL1*. The clinical assessment of the mother revealed that she had no significant autistic symptoms (SRS total scores below the 66th centile), no axis I psychiatric comorbidities (using the DIGS) and no significant medical history. The clinical exam was also in the normal range although she shared the same signs of dysmorphism with her daughter. The mother reported that two of her three brothers (subjects II-C & II-E) had severe learning disabilities. The third one (subject II-D) seemed to have no personal history of ID, speech delay or learning disability, but his son (seven years old) had a significant language delay and needed speech therapy (subject III-J). Unfortunately we were unable to explore the full segregation of the CNVs in the siblings of the mother since only one of them was available at time of the inclusion for clinical and genetic investigations.

We also investigated the sisters of the proband (subject III-A, III-B, III-C, III-E and III-F). Only one (III-C) had normal IQ (Full Scale IQ=92) and presented normal speech development. Others presented either moderate speech delay with first sentences at 4 years (III-A, III-B), or both speech disorder and mild to moderate ID (III-E, III-F). One of the two affected maternal uncles (Subject II-C, Figure 5) was assessed for clinical and genetic screening and had severe ID and speech disorder using only few words. He attended school until about 10 years old, and works in an integration center for persons with mental health problems. He had two unaffected daughters and one boy who presented with ID and speech delay. He was not available for formal neuro-evaluation and genetic testing.

**SUPPLEMENTARY REFERENCES**

1. Phelan, K. & McDermid, H. E. The 22q13.3 Deletion Syndrome (Phelan-McDermid Syndrome). *Mol. Syndromol.* **2,** 186–201 (2012).

2. Weiss, L., Shen, Y. & Korn, J. Association between Microdeletion and Microduplication at 16p11.2 and Autism. *N. Engl. J. Med.* **358,** 2255–2265 (2014).

3. Fernandez, B. A. *et al.* Phenotypic spectrum associated with de novo and inherited deletions and duplications at 16p11.2 in individuals ascertained for diagnosis of autism spectrum disorder. *J. Med. Genet.* **47,** 195–203 (2010).

4. Bochukova, E. G. *et al.* Large, rare chromosomal deletions associated with severe early-onset obesity. *Nature* **463,** 666–670 (2010).

5. Walters, R. G. *et al.* A new highly penetrant form of obesity due to deletions on chromosome 16p11.2. *Nature* **463,** 671–5 (2010).

6. Abdelmoity, A. T. *et al.* 15Q11.2 Proximal Imbalances Associated With a Diverse Array of Neuropsychiatric Disorders and Mild Dysmorphic Features. *J. Dev. Behav. Pediatr.* **33,** 570–6 (2012).

7. Ullmann, R. *et al.* Array CGH Identifies Reciprocal 16p13 . 1 Duplications and Deletions That Predispose to Autism and / or Mental Retardation. *Hum. Mutat.* **28,** 674–682 (2007).

8. Hannes, F. D. *et al.* Recurrent reciprocal deletions and duplications of 16p13.11: the deletion is a risk factor for MR/MCA while the duplication may be a rare benign variant. *J. Med. Genet.* **46,** 223–32 (2009).

9. Ingason, A. *et al.* Copy number variations of chromosome 16p13.1 region associated with schizophrenia. *Mol Psychiatry* **16,** 17–25 (2009).

10. Tropeano, M. *et al.* Male-Biased Autosomal Effect of 16p13.11 Copy Number Variation in Neurodevelopmental Disorders. *PLoS One* **8,** e61365 (2013).

11. Vanlerberghe, C. *et al.* 15q 11.2 microdeletion (BP1-BP2) and developmental delay, behaviour issues, epilepsy and congenital heart disease: A series of 52 patients. *Eur. J. Med. Genet.* **58,** 140–147 (2015).

12. Cafferkey, M., Ahn, J. W., Flinter, F. & Ogilvie, C. Phenotypic features in patients with 15q11.2(BP1-BP2) deletion: Further delineation of an emerging syndrome. *Am. J. Med. Genet. Part A* **164,** 1916–1922 (2014).

13. Cox, D. M. & Butler, M. G. The 15q11.2 BP1-BP2 microdeletion syndrome: A review. *Int. J. Mol. Sci.* **16,** 4068–4082 (2015).

14. Chaste, P. *et al.* Modest impact on risk for autism spectrum disorder of rare copy number variants at 15q11.2, Specifically Breakpoints 1 to 2. *Autism Res.* **7,** 355–362 (2014).

15. Das, D. K. *et al.* Genetic and morphological features of human iPSC-derived neurons with chromosome 15q11.2 (BP1-BP2) deletions. *Mol. neuropsychiatry* **1,** 116–123 (2015).
