# Supplementary figures and images for: A framework to identify contributing genes in patients with Phelan-McDermid syndrome
Source: NPJ Genom Med. 2017 Oct 23;2:32. doi: 10.1038/s41525-017-0035-2 (PMC5677962; doi:10.1038/s41525-017-0035-2)

Normal corpus callosum

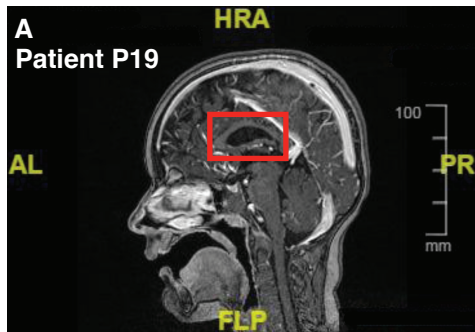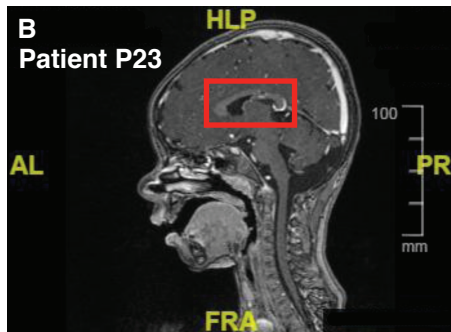

Abnormal corpus callosum

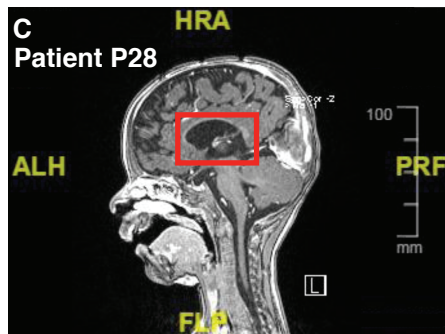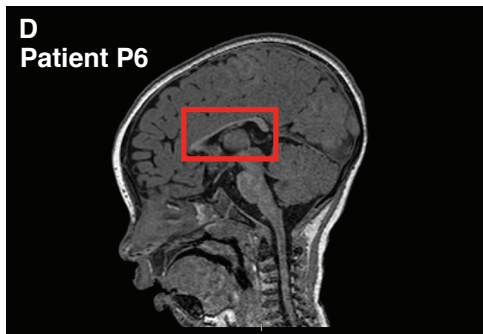

Supplement: Supplementary file 2 — Supplementary Figure 1 [file 41525_2017_35_MOESM2_ESM.pdf]

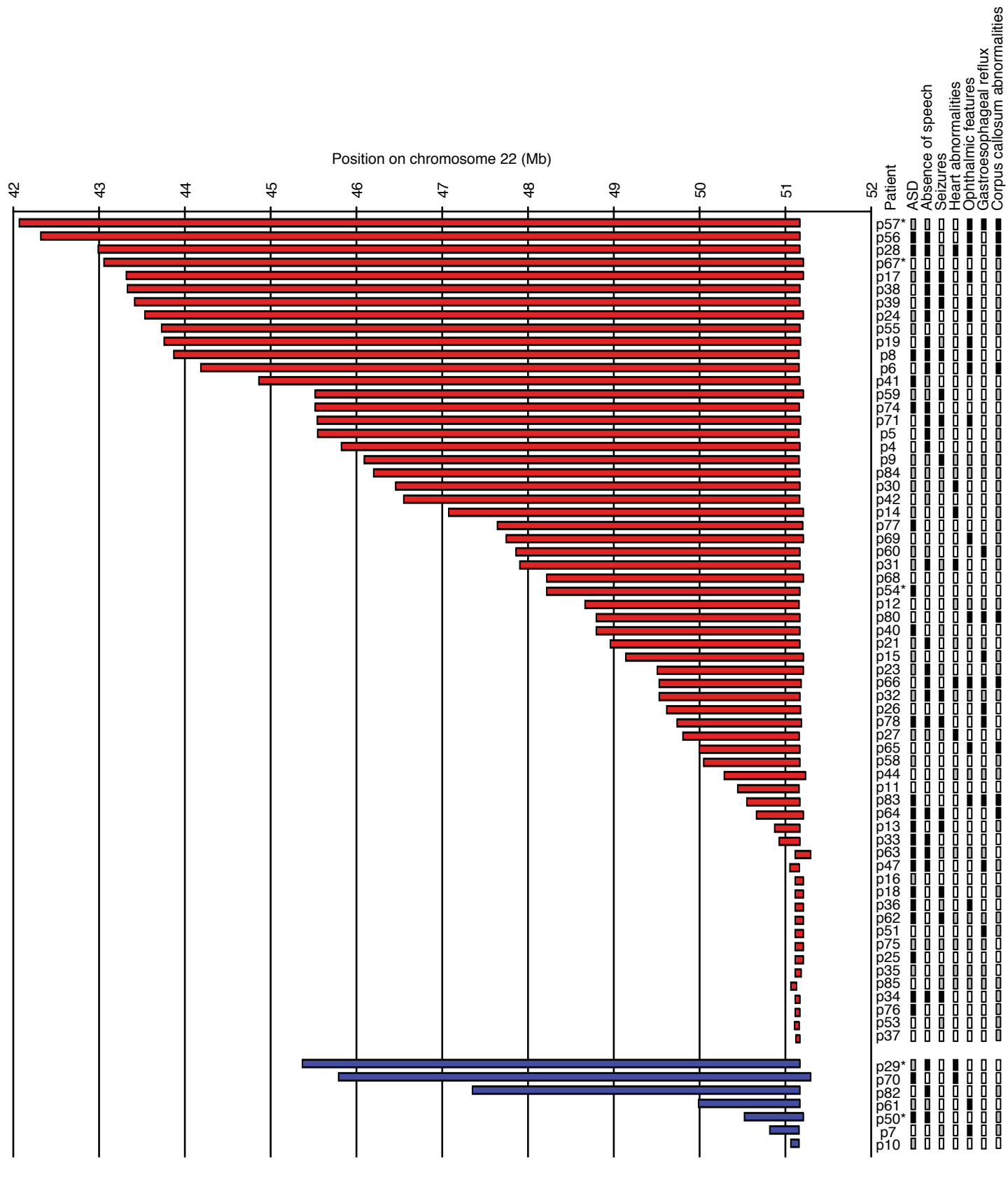

Supplement: Supplementary file 3 — Supplementary Figure 2 [file 41525_2017_35_MOESM3_ESM.pdf]

# A

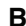

B

Supplement: Supplementary file 5 — Supplementary Figure 4 [file 41525_2017_35_MOESM5_ESM.pdf]

### Small CNVs

### Small CNVs incl. NP-genes

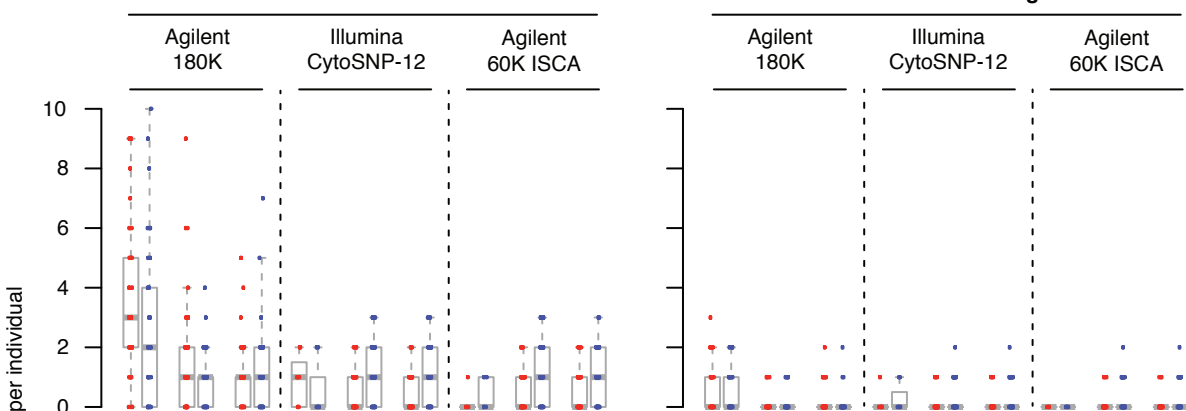

### Large CNVs

### Large CNVs incl. NP-genes

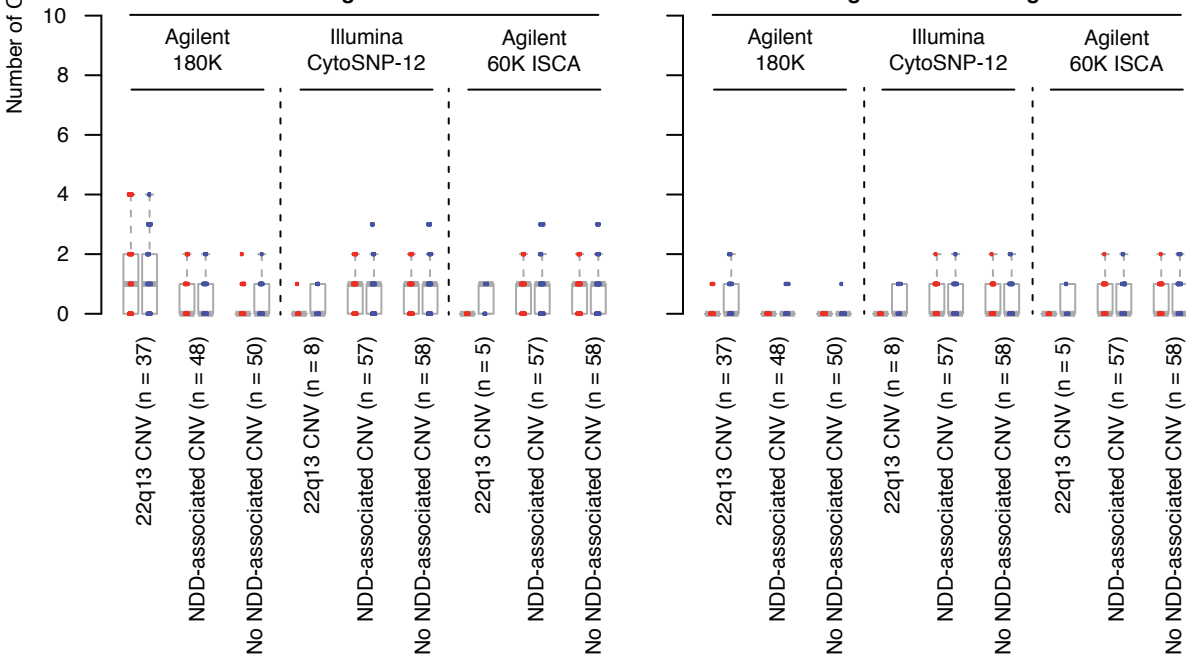

• Deletions

• Duplications

Supplement: Supplementary file 6 — Supplementary Figure 5 [file 41525_2017_35_MOESM6_ESM.pdf]

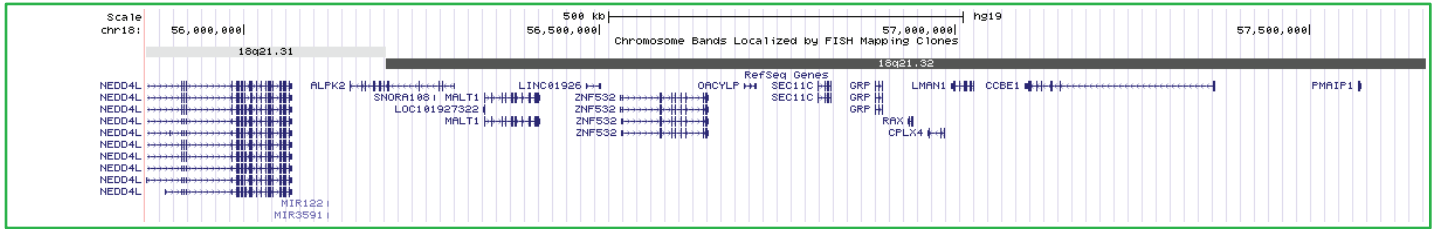

Supplement: Supplementary file 8 — Supplementary Figure 7 [file 41525_2017_35_MOESM8_ESM.pdf]

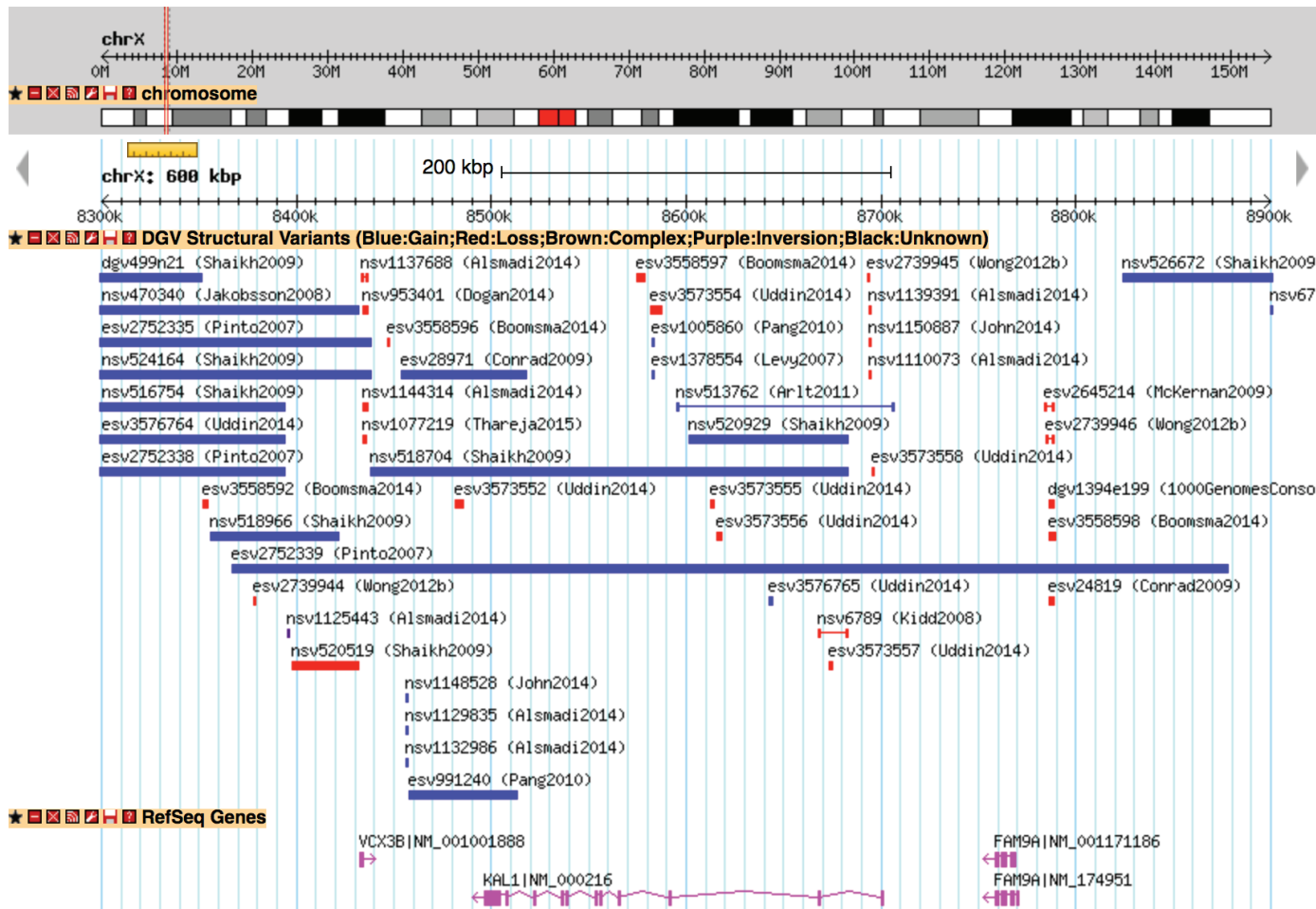

Supplement: Supplementary file 10 — Supplementary Figure 9 [file 41525_2017_35_MOESM10_ESM.pdf]

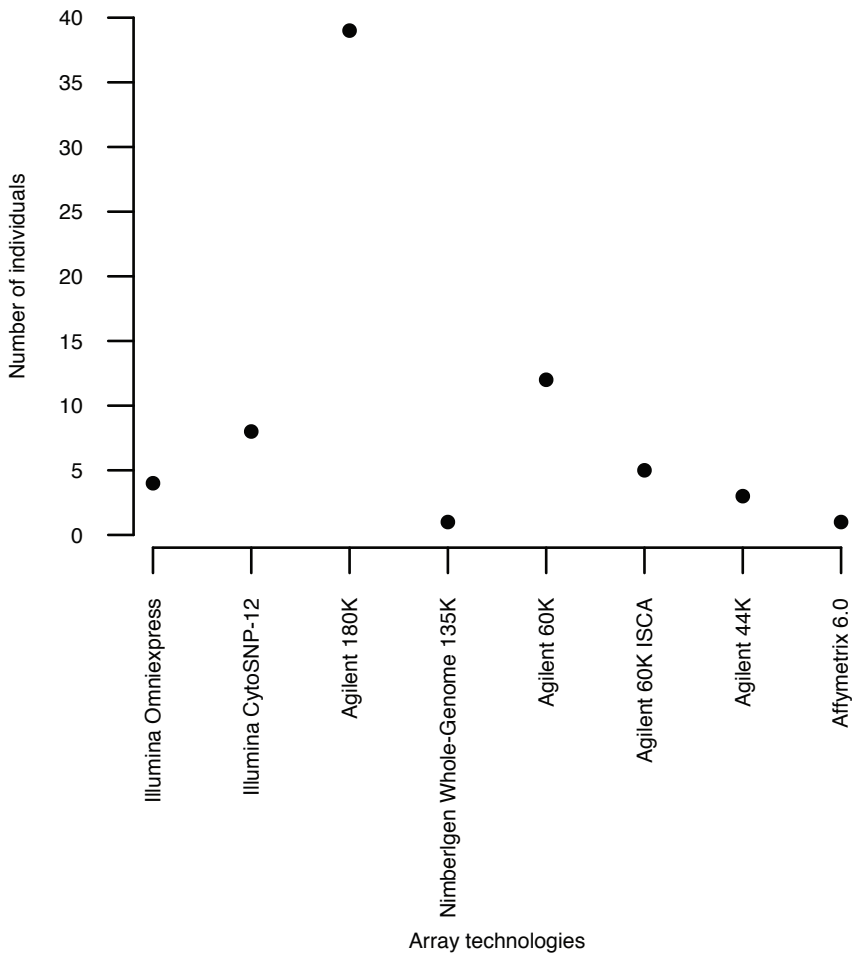

Supplement: Supplementary file 11 — Supplementary Figure 10 [file 41525_2017_35_MOESM11_ESM.pdf]

# Hapmap3

## Africa

- ASW
- LWK
- MKK
- YRI

## Europe

- CEU
- TSI

## Asia

- CHB
- CHD
- JPT

- GIH
- MEX

- This work

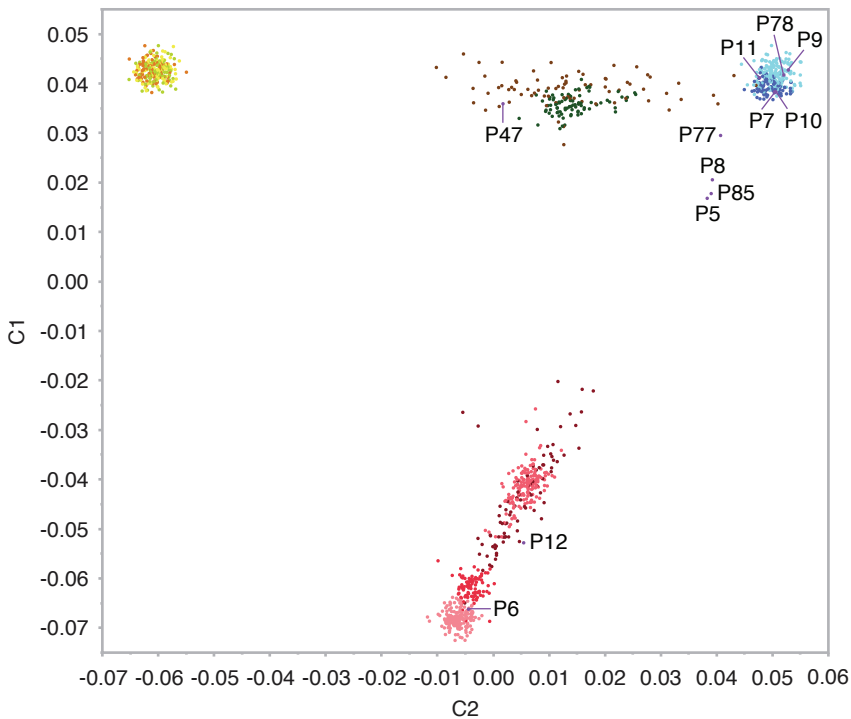

Supplement: Supplementary file 12 — Supplementary Figure 11 [file 41525_2017_35_MOESM12_ESM.pdf]
