# Supplementary material for: A framework to identify contributing genes in patients with Phelan-McDermid syndrome
Source: NPJ Genom Med. 2017 Oct 23;2:32. doi: 10.1038/s41525-017-0035-2 (PMC5677962; doi:10.1038/s41525-017-0035-2)
Supplement: Supplementary file 4 — Supplementary Figure 3 [file 41525_2017_35_MOESM4_ESM.pdf]

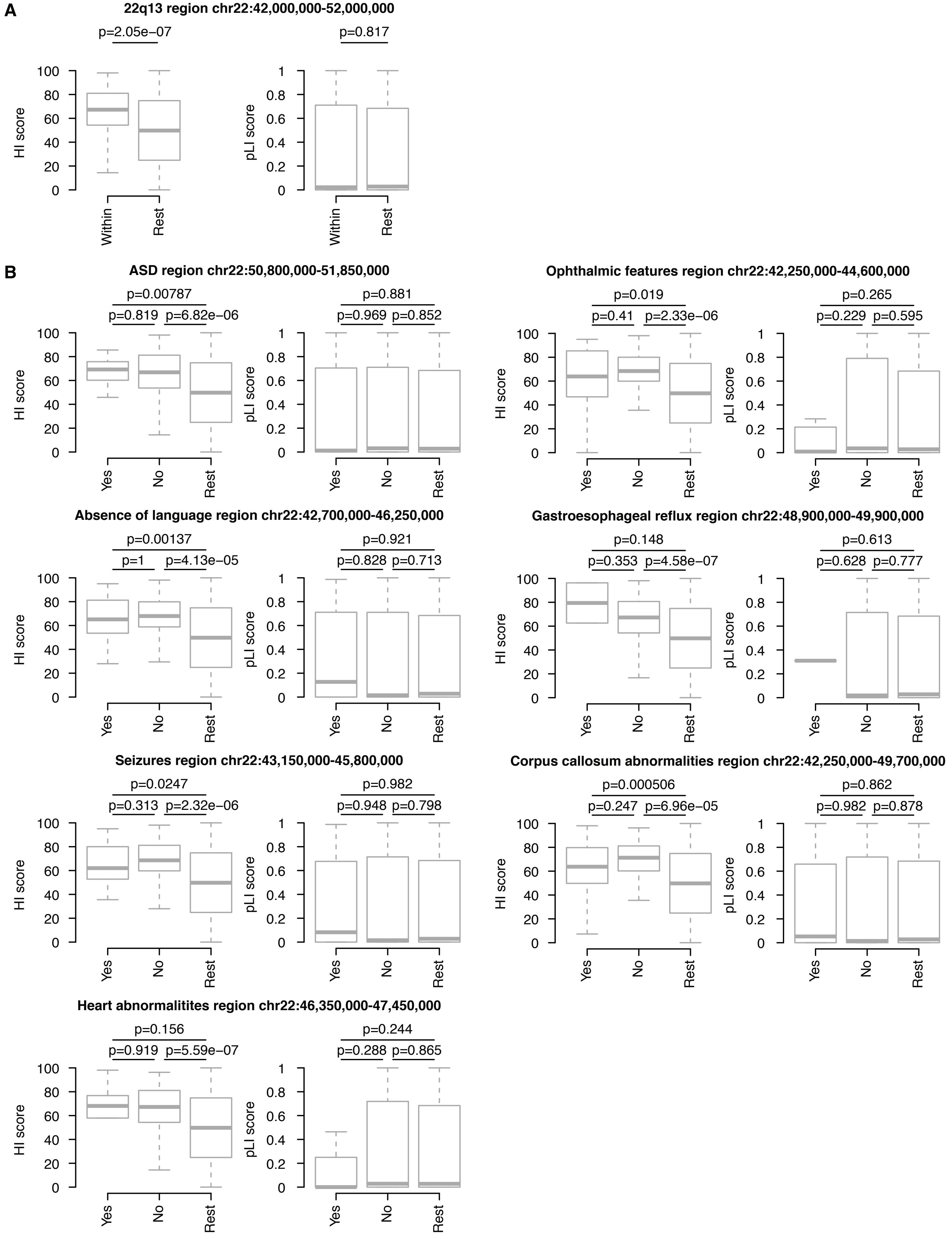
A
22q13 region chr22:42,000,000-52,000,000
p=2.05e−07
p=0.817
HI score
pLI score
Within
Rest
B
ASD region chr22:50,800,000-51,850,000
p=0.00787
p=0.819
p=6.82e−06
p=0.881
p=0.969
p=0.852
Ophthalmic features region chr22:42,250,000-44,600,000
p=0.019
p=0.41
p=2.33e−06
p=0.265
p=0.229
p=0.595
Absence of language region chr22:42,700,000-46,250,000
p=0.00137
p=1
p=4.13e−05
p=0.921
p=0.828
p=0.713
Gastroesophageal reflux region chr22:48,900,000-49,900,000
p=0.148
p=0.353
p=4.58e−07
p=0.613
p=0.628
p=0.777
Seizures region chr22:43,150,000-45,800,000
p=0.0247
p=0.313
p=2.32e−06
p=0.982
p=0.948
p=0.798
Corpus callosum abnormalities region chr22:42,250,000-49,700,000
p=0.000506
p=0.247
p=6.96e−05
p=0.862
p=0.982
p=0.878
Heart abnormalitites region chr22:46,350,000-47,450,000
p=0.156
p=0.919
p=5.59e−07
p=0.244
p=0.288
p=0.865
Yes
No
Rest
