# Supplementary material for: A framework to identify contributing genes in patients with Phelan-McDermid syndrome
Source: NPJ Genom Med. 2017 Oct 23;2:32. doi: 10.1038/s41525-017-0035-2 (PMC5677962; doi:10.1038/s41525-017-0035-2)
Supplement: Supplementary file 7 — Supplementary Figure 6 [file 41525_2017_35_MOESM7_ESM.pdf]

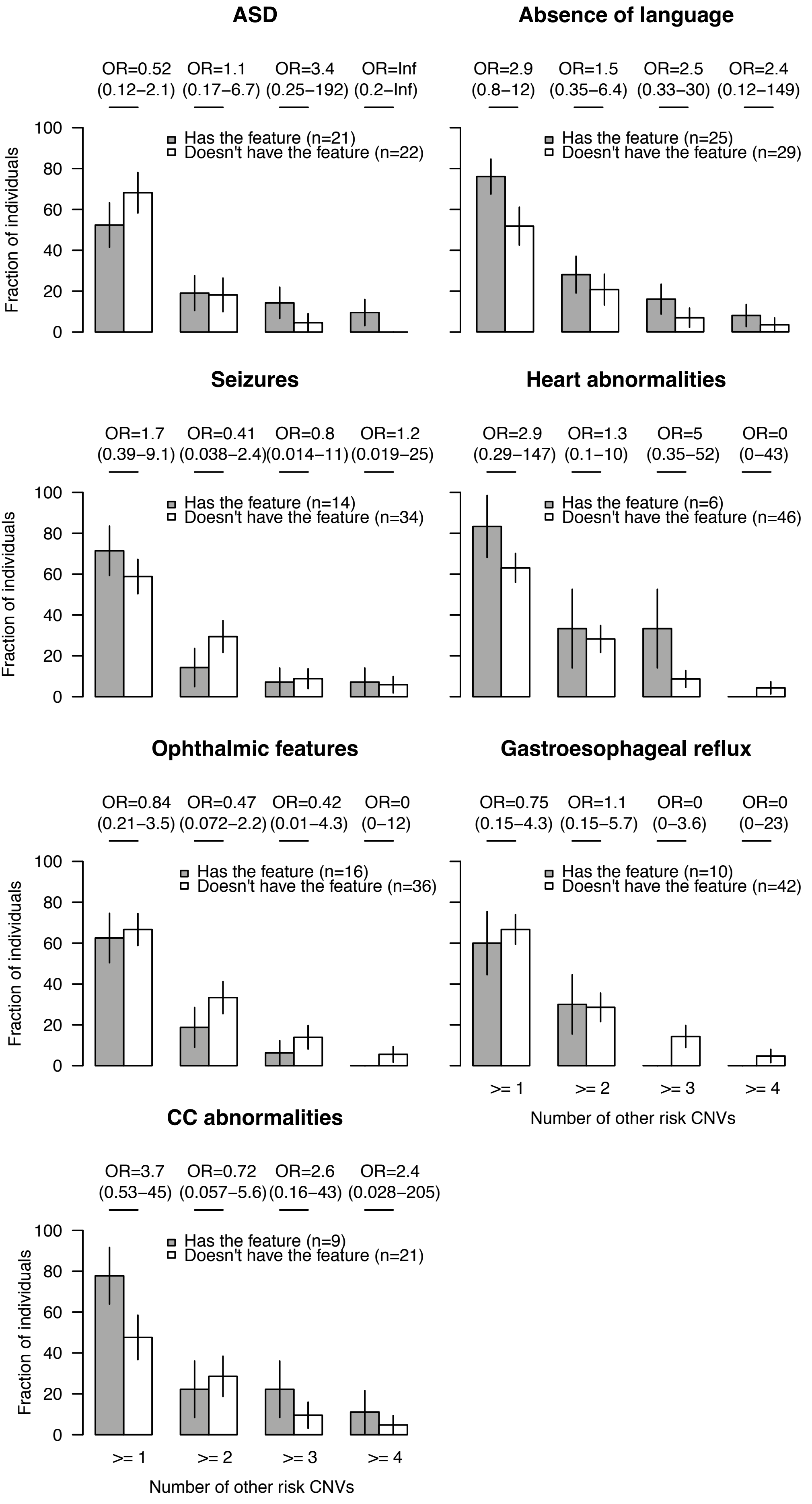
ASD
OR=0.52 (0.12–2.1)
OR=1.1 (0.17–6.7)
OR=3.4 (0.25–192)
OR=Inf (0.2–Inf)
Has the feature (n=21)
Doesn't have the feature (n=22)
Absence of language
OR=2.9 (0.8–12)
OR=1.5 (0.35–6.4)
OR=2.5 (0.33–30)
OR=2.4 (0.12–149)
Has the feature (n=25)
Doesn't have the feature (n=29)
Seizures
OR=1.7 (0.39–9.1)
OR=0.41 (0.038–2.4)
OR=0.8 (0.014–11)
OR=1.2 (0.019–25)
Has the feature (n=14)
Doesn't have the feature (n=34)
Heart abnormalities
OR=2.9 (0.29–147)
OR=1.3 (0.1–10)
OR=5 (0.35–52)
OR=0 (0–43)
Has the feature (n=6)
Doesn't have the feature (n=46)
Ophthalmic features
OR=0.84 (0.21–3.5)
OR=0.47 (0.072–2.2)
OR=0.42 (0.01–4.3)
OR=0 (0–12)
Has the feature (n=16)
Doesn't have the feature (n=36)
Gastroesophageal reflux
OR=0.75 (0.15–4.3)
OR=1.1 (0.15–5.7)
OR=0 (0–3.6)
OR=0 (0–23)
Has the feature (n=10)
Doesn't have the feature (n=42)
CC abnormalities
OR=3.7 (0.53–45)
OR=0.72 (0.057–5.6)
OR=2.6 (0.16–43)
OR=2.4 (0.028–205)
Has the feature (n=9)
Doesn't have the feature (n=21)
Fraction of individuals
0
20
40
60
80
100
>= 1
>= 2
>= 3
>= 4
Number of other risk CNVs
