# Supplementary material for: A framework to identify contributing genes in patients with Phelan-McDermid syndrome
Source: NPJ Genom Med. 2017 Oct 23;2:32. doi: 10.1038/s41525-017-0035-2 (PMC5677962; doi:10.1038/s41525-017-0035-2)
Supplement: Supplementary file 9 — Supplementary Figure 8 [file 41525_2017_35_MOESM9_ESM.pdf]

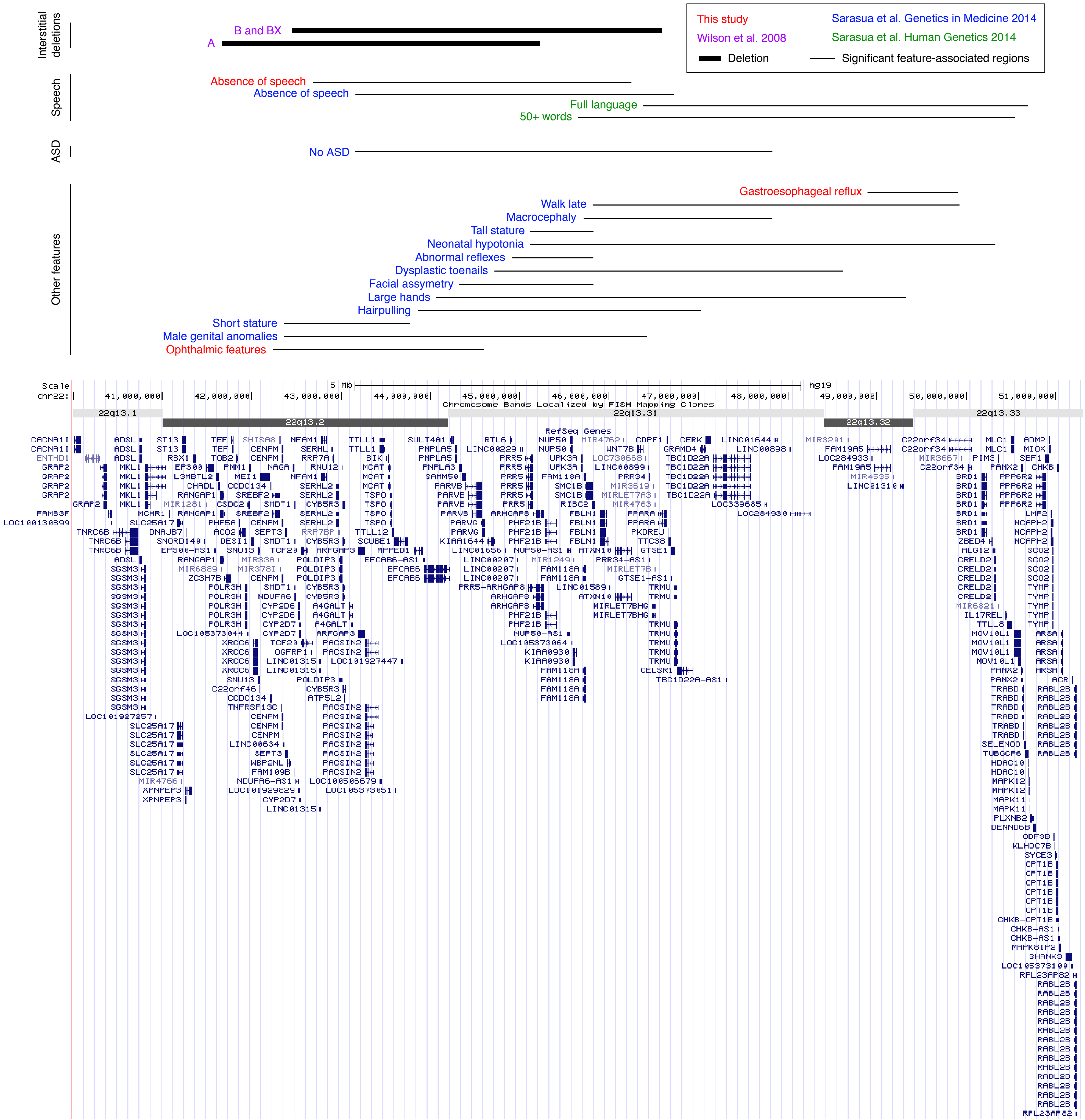

Interstitial deletions
B and BX
A
This study
Sarasua et al. Genetics in Medicine 2014
Wilson et al. 2008
Sarasua et al. Human Genetics 2014
Deletion
Significant feature-associated regions
Speech
Absence of speech
Absence of speech
Full language
50+ words
ASD
No ASD
Other features
Gastroesophageal reflux
Walk late
Macrocephaly
Tall stature
Neonatal hypotonia
Abnormal reflexes
Dysplastic toenails
Facial assymetry
Large hands
Hairpulling
Short stature
Male genital anomalies
Ophthalmic features
Scale
5 Mb
hg19
chr22:
41,000,000
42,000,000
43,000,000
44,000,000
45,000,000
46,000,000
47,000,000
48,000,000
49,000,000
50,000,000
51,000,000
Chromosome Bands Localized by FISH Mapping Clones
22q13.1
22q13.2
22q13.31
22q13.32
22q13.33
RefSeq Genes
